# Blending PLA with Polyesters Based on 2,5-Furan Dicarboxylic Acid: Evaluation of Physicochemical and Nanomechanical Properties

**DOI:** 10.3390/polym14214725

**Published:** 2022-11-04

**Authors:** Zoi Terzopoulou, Alexandra Zamboulis, Lazaros Papadopoulos, Maria-Eirini Grigora, Konstantinos Tsongas, Dimitrios Tzetzis, Dimitrios N. Bikiaris, George Z. Papageorgiou

**Affiliations:** 1 Department of Chemistry, University of Ioannina, GR-45110 Ioannina, Greece; 2 Laboratory of Polymer and Colors Chemistry and Technology, Department of Chemistry, University of Thessaloniki, GR-54124 Thessaloniki, Greece; 3 Digital Manufacturing and Materials Characterization Laboratory, School of Science and Technology, International Hellenic University, 14km, N. Moudania, GR-57001 Thessaloniki, Greece; 4 Institute of Materials Science and Computing, University Research Center of Ioannina (URCI), GR-45110 Ioannina, Greece

**Keywords:** polymer blends, poly(lactic acid), aliphatic-aromatic copolyesters, 2,5-furan dicarboxylic acid

## Abstract

Poly(lactic acid) (PLA) is a readily available, compostable biobased polyester with high strength and toughness, and it is excellent for 3D printing applications. Polymer blending is an economic and easy way to improve its properties, such as its slow degradation and crystallization rates and its small elongation, and thus, make it more versatile. In this work, the effects of different 2,5-furan dicarboxylic acid (FDCA)-based polyesters on the physicochemical and mechanical properties of PLA were studied. Poly(butylene furan 2,5-dicarboxylate) (PBF) and its copolymers with poly(butylene adipate) (PBAd) were synthesized in various comonomer ratios and were blended with 70 wt% PLA using melt compounding. The thermal, morphological and mechanical properties of the blends are investigated. All blends were immiscible, and the presence of the dispersed phases improved the crystallization ability of PLA. Mechanical testing revealed the plasticization of PLA after blending, and a small but measurable mass loss after burying in soil for 7 months. Reactive blending was evaluated as a compatibilizer-free method to improve miscibility, and it was found that when the thermal stability of the blend components allowed it, some transesterification reactions occurred between the PLA matrix and the FDCA-based dispersed phase after 20 min at 250 °C.

## 1. Introduction

Poly(lactic acid) (PLA) is a biobased and compostable aliphatic polyester that has been studied for use in many applications over the last decade. It is currently the most widely used biobased polymer in the world, and due to its excellent printability in 3D printing, its demand exceeds production. Many properties of PLA, such as strength, stiffness and gas permeability, were found to be comparable to those of traditional petrochemical-based polymers. However, PLA-based materials have a significant number of limitations for specific applications, such as slow biodegradation, high cost and low hardness [1]. The modification of PLA by blending it with other polymers to achieve suitable properties for different applications has received considerable attention in recent years. When compared with copolymerization, polymer blending is an easier and more cost-effective method of fabricating polymer-based materials for a wide range of applications.

Polymer blends containing PLA are offered in the market by several companies, including Bioflex^®^ from FKuR Kunststoff GmbH (Willich, Germany) and Ecovio^®^ from BASF (Ludwigshafen, Germany) [2]. Ecovio^®^ is a compostable, partially biobased blend of PLA with petroleum-based poly(butylene adipate-co-terephthalate) (PBAT) that is suitable for the fabrication of bags and mulch films. The addition of PBAT improves the elongation and toughness of PLA.

An integral part of the efforts to transition to a more sustainable society is the development and scaling up of the production of new biobased polymers. 2,5-Furan dicarboxylic acid (FDCA)-based polyesters and poly(ethylene 2,5-furan dicarboxylate) (PEF) in particular are expected to play a dominant role in the ever-growing biobased plastics market [3,4,5,6,7]. PEF is considered one of the most promising biobased polymers to replace the petroleum-based poly(ethylene terephthalate), either fully or partially [3]. Avantium has already started constructing a new plant with the capacity to produce 5 kilotonnes of FDCA per year, which is expected to reduce the price of biomass-derived FDCA significantly and, therefore, make the production of cost-competitive PEF possible as well. Besides PEF, a plethora of polyesters of FDCA and their copolymers were reported, with a wide range of properties that can be tuned by tuning their composition [6,8,9]. In the efforts to impart biodegradability to FDCA homopolyesters, adipic acid was used. PBF-co-poly(butylene adipate) (PBAd) copolymers are compostable and can behave either as thermoplastics or as elastomers, depending on the comonomer composition [10,11,12,13,14].

Because of the sustainable character, as well as the good barrier and mechanical properties of FDCA-based polyesters, the number of reports in the literature concerning their blends with PLA is increasing [15,16,17,18,19,20,21,22,23]. In our previous work, we prepared blends with PLA and PEF, poly(propylene 2,5-furan dicarboxylate) (PPF), or poly(butylene 2,5-furan dicarboxylate (PBF) using solution casting [20,23]. These blends were immiscible in all compositions tested. Regardless of the immiscibility, Long et al. [22] found that uncompatibilized PBF/PLA blends with small PBF content showed that the elongation of the blends was 17 times greater than PLA, while the measure of elasticity and the strength at the breaking point remained the same. In addition, impact resistance was improved compared with the two neat polymers. This peculiar behavior was attributed to the glass–amorphous transition and stretch-induced crystallization in the PBF phase. Uncompatibilized poly(alkylene 2,5-furan dicarboxylate) (PAF)/PLA, including PBF/PLA blends prepared with solvent casting, had improved elongation in comparison with neat PLA [15]. Blending poly(pentylene 2,5-furan dicarboxylate) (PPeF) with PLA improved its UV-shielding properties, its barrier properties and its ductility despite their immiscibility [19]. Additionally, blends of PLA with PPeF, POF and PDoF are suitable for producing textile fibers using wet-spinning [18,21].

The scope of this work was to prepare PLA/PBF and PLA/PBF-co-PBAd blends using melt blending and evaluate their physicochemical properties. The miscibility was assessed with SEM and DSC, while mechanical properties were tested with nanoindentation testing. Nanoindentation tests were assisted by a finite element analysis (FEA) process to curve fit the experimental load–depth curves and extract the materials’ stress–strain behavior. Structural properties were characterized with FTIR and XRD and the effect of the blending on the soil degradation of PLA was studied using mass loss quantification and surface observation with microscopy. Finally, reactive blending was tested as a compatibilizer-free approach to improve blend miscibility.

## 2. Materials and Methods

### 2.1. Materials

The PLA used was Ingeo^TM^ Biopolymer 3052D (NatureWorks, Plymouth, MN, USA), which was designed for injection molding [24], and it was kindly donated by Plastika Kritis S.A., Heraklion, Greece. It contains ~96% of L- and ~4% of D-lactide and has Mn = 81,700 g/mol. Its intrinsic viscosity is [*η*] = 1.24 dL/g.

### 2.2. Synthesis of the Polyesters

For the synthesis of PBAd, the following protocol was used: First, for the esterification step, adipic acid and 1,4-butanediol in a molar ratio 1:1.1 were charged into a three-necked round bottom flask equipped with a condenser, a mechanical stirrer and a nitrogen inlet. The mixture was gradually heated until homogenization, and then the temperature was increased to 190 °C for 4 h. Then, for the polycondensation step, 400 ppm TBT was added to the reaction mixture, and a vacuum of 0.05 mbar was applied gradually over 30 min. The reaction temperature was increased to 220 °C for an additional 3 h.

For the synthesis of PBF, the following protocol was used: First, for the transesterification step, 2,5-dimethyl furan dicarboxylate (DMFD) and 1,4-butanediol in a molar ratio 1:2.2 were charged into the apparatus that was previously described. Furthermore, 400 ppm of TBT was added to act as a catalyst for transesterification. The mixture was gradually heated until homogenization, and then the temperature was set at 160 °C for 1 h, 170 °C for 1 h, 180 °C for another hour and, finally, 190 °C for an additional hour. For the polycondensation step, a vacuum of 0.05 mbar was applied over 30 min and the reaction temperature was set to 210 °C for 1 h, 220 °C for 1 h and 230 °C for 1 h.

For the synthesis of the copolymers, the product from the esterification step of PBAd synthesis, and the product from the transesterification step of PBF synthesis were used. 6-(4-Hydroxybutoxy)-6-oxohexanoic acid and bis(4-hydroxylbutyl) furan 2,5-dicarboxylate at molar ratios of 3:1, 1:1 and 1:3 were charged into the previously described apparatus. Then, 400 ppm of TBT was added to the flask, and the temperature was set at 160 °C for 1 h, 170 °C for 1 h, 180 °C for 1 h and 190 °C for an additional hour. For the polycondensation step, a vacuum of 0.05 mbar was applied over 30 min and the reaction temperature was set to 210 °C for 1 h, 220 °C for 1 h and 230 °C for 1 h. Through this protocol, the materials PBF-PBAd 75-25, PBF-PBAd 50-50 and PBF-PBAd 25-75 were synthesized.

### 2.3. Blend Preparation

Blends containing 70 wt% PLA and 30 wt% of each FDCA-based polyester were prepared via melt blending. Prior to this, the components were dried overnight under a vacuum. To prepare the blends, the components were introduced in a twin screw co-rotating extruder, operating at 190 °C and 35 rpm for 5 min.

### 2.4. Characterization

The intrinsic viscosity of the produced polyester was measured with an Ubbelohde viscometer (Schott Gerate GMBH, Hofheim, Germany) at 25 °C using a phenol/1,1,2,2-tetrachloroethane (60/40 *w*/*w*) solution. The sample was heated in the solvent mixture at 80 °C for 20 min until complete dissolution. After cooling, the solution was filtered through a disposable Teflon filter to remove possible solid residues. The calculation of the intrinsic viscosity value of the polymer was performed by applying the Solomon–Cuita Equation (1) of a single point measurement:

(1) [η]=[2{tt0−ln(tt0)−1}]12c,

where *c* is the solution concentration, *t* is the flow time of the solution and *t*_0_ is the flow time of the solvent. The experiment was performed three times and the average value was estimated.

Molecular weight was measured with an Agilent Technologies 1260 Infinity II LC Gel Permeation Chromatography (GPC) System consisting of an Isocratic Pump, a PL gel MIXED Guard column and two PLgel 5 μm MIXED-C columns, and an Agilent RID detector. For the calibration, 10 polystyrene (PS) standards of molecular weights between 600 and 1,000,000 g/mol were employed. The prepared solutions had a concentration of 1 mg/mL and the injection volume was 25 μL with a flow of 1 mL/min at a temperature of 40 °C.

Nuclear magnetic resonance (NMR) spectra were recorded in deuterated chloro-form for the structural study of the synthesized polymers. An Agilent 500 spectrometer was utilized (Agilent Technologies, Santa Clara, CA, USA) at room temperature. Spectra were calibrated using the residual solvent peaks.

The morphology of cryofractured cross-sections of the samples was studied with a JEOL (Tokyo, Japan) JSM 7610F field emission scanning electron microscope (SEM) operating at 5 kV.

ATR spectra of the samples were recorded using an IRTracer-100 (Shimadzu, Kyoto, Japan) equipped with a QATR™ 10 Single-Reflection ATR Accessory with a Diamond Crystal. The spectra were collected in the range from 450 to 4000 cm^−1^ at a resolution of 2 cm^−1^ (a total of 16 co-added scans), while the baseline was corrected and converted into absorbance mode.

XRD diffractograms were recorded using a MiniFlex II XRD system (Rigaku, Co., Tokyo, Japan) with Cu Kα radiation (0.154 nm) over the 2θ range from 5° to 50° with a scanning rate of 1°/min. Melt-quenched films prepared with compression molding that were first annealed at the peak of their T_cc_ for 1 h were used.

Differential scanning calorimetry (DSC) analysis was performed using a Perkin Elmer Pyris Diamond DSC differential scanning calorimeter (Solingen, Germany) calibrated with pure indium and zinc standards. The system included a PerkinElmer Intracooler 2 (Solingen, Germany) cooling accessory. Samples of 5 ± 0.1 mg sealed in aluminum pans were used to test the thermal behavior of the polymers.

Thermogravimetric analysis (TGA) was carried out using a SETARAM (Caluire, France) SETSYS TG-DTA 16/18 instrument. The samples (6 ± 0.5 mg) were placed in alumina crucibles and heated from 25 °C to 600 °C in a 50 mL/min flow of N_2_ at heating rates of 20 °C/min.

Mechanical properties measurements were performed at room temperature (25 °C). The nanoindentation measurements were carried out on a DUH-211S Shimadzu (Kyoto, Japan) device with a force resolution of 0.196 μN. A diamond triangular tip Berkovich indenter (angle of 65°, tip radius is 100 nm) was used and three points were selected and measured using an optical microscope integrated into the device. The modulus and hardness were determined based on the method of Oliver and Pharr [25] and previous work [26,27,28,29,30]. The maximum load was 10 mN and was achieved at a rate of 1.66 mN/s. Due to the material’s viscoelastic nature, a dwell time of 50 s was implemented to allow for sufficient time at peak load for the creep effects to saturate. The additional depth induced during the dwell time at constant load was recorded to provide insight into the creep response of the material. In order to calculate the nanomechanical properties, the average value of ten measurements taken at different locations was used. A finite element analysis (FEA) process was developed in order to fit the nanoindentation test curves and extract the stress–strain behavior of the specimens. The interface between the indenter and the surface of the sample was simulated with contact elements and assumed to be frictionless. The nanoindentation experiments were computationally generated by considering the simulation of the loading stage of the indenter penetrating the surface. Other works [26,27,31] showed that kinematic hardening leads to a rapid convergence in the corresponding FEA calculations, and thus, this method was utilized in the developed curve-fitting procedure.

The water contact angle was measured with an Ossila (Sheffield, United Kingdom) contact angle goniometer L2004A1 at room temperature (25 °C). The contact angle was measured by gently placing a water droplet (5 µL) on the surface of the films of the samples prepared via compression molding. At least three measurements were performed and the mean value is reported herein.

For the preliminary evaluation of degradability in soil, the samples were buried in a pot containing a 1:1 by weight mixture of soil and digested sheep manure at an outdoors temperature in Thessaloniki, Greece, during March to September 2021. The soil was collected from a horticulture farm in the rural region of Thessaloniki (40°40′35.3″ N 23°15′37.9″ E). The climate of Thessaloniki is Mediterranean, with hot and dry summers and warm and temperate springs. The average daily temperature exported from a local weather station is presented in Figure S1. Moisture in the soil was replenished at regular intervals with tap water. After predetermined time intervals, the samples were removed from the soil, dried under a vacuum for 3 days and weighed.

The mass loss percentage was calculated according to the following equation:

(2) $$Mass\;loss\;\%=\frac{W_{0}-W_{i}}{W_{0}},$$

where *w*_0_ is the initial sample of the sample and *w_i_* is the final weight after a certain time interval.

Each sample was buried in triplicate and the mean value was calculated. The surface of the samples was examined using a Jenoptik (Jena, Germany) ProgRes GRYPHAX ARKTUR camera attached to a ZEISS (Oberkochen, Germany) SteREO Discovery V20 microscope, and Gryphax image capturing software and the scanning electron microscope described above were also used.

## 3. Results

### 3.1. Synthesis of the FDCA-Based Polyesters

**Scheme 1:**
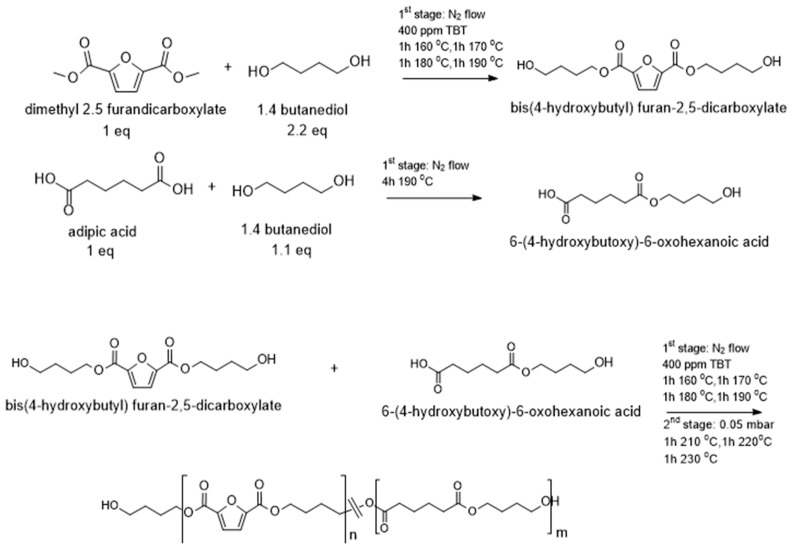
Synthesis of PBF, PBAd and their copolymers.

The synthetic procedure applied is presented in Scheme 1 and the obtained molecular intrinsic viscosity, Mn and PDI are shown in Table 1. Butylene adipate (BAd) and butylene furanoate (BF) oligomers were first prepared, and then each one was added in the proper amounts to produce the copolymers in question through polycondensation. The use of dihydroxybutylene furanoate and dihydroxybutylene adipate assured that the monomer ratio was very close to the feed ratio, as only 1,4-butanediol molecules can be removed during the polycondensation step. Furthermore, materials of high molecular weight up to 23,000 g/mol were obtained, confirming that the protocol followed was suitable for the synthesis of copolyesters. Three materials with different compositions were prepared to examine the effect of each comonomer on the properties of the resulting material.

The NMR spectra of the copolymers are presented in Figure S2. For the PBF-PBAd copolymers, resonance signals corresponding to the BF segments were observed at 7.28 ppm CH C, 4.44 ppm CH_2_ D and 1.92 ppm CH_2_ E. The BAd peaks were observed at 4.15 ppm OCH_2_ 4, 2.41 ppm CH_2_C(O) 2, 1.73 ppm CH_2_ 5 and 1.65 ppm CH_2_ 3. Additionally, signals corresponding to butanol units linked to both FDCA and adipic acids were also observable: 4.41 ppm OCH_2_ D′, 4.15 ppm OCH_2_ 4′, 1.85 ppm E′ and 1.80 ppm 5′ (Figure S2c). The ^13^C spectra (Figure S2b) confirmed the ^1^H NMR spectra. Two ester peaks were observed at 174.5 (C=O 1) and 158.3 (C=O A) ppm, along with all the other expected signals: 146.5 and 118.8 ppm CH B and C, 65.1 ppm OCH_2_ D, 64.3 ppm OCH_2_ 4, 33.8 ppm CH_2_C(O) 2, 25.01 ppm CH_2_ E, 24.98 ppm CH_2_ 5 and 24.2 ppm CH_2_ 3.

The composition of the copolymers was calculated using the integrations of the peaks at 7.28 ppm and 2.41 ppm (Table 2). The microstructure of the copolymers was deduced from peaks at 4.44 to 4.15 ppm corresponding to the butanol methylene groups adjacent to the ester linkages. As depicted in Figure S3, there were three possible structures according to the acid that is linked to each side of butanol: FBF, FBAd (equivalent to AdBF) and AdBAd. The corresponding resonance signals (Figure S2c) were used to calculate the average sequence length of the butylene furanoate (*L_BF_*) and butylene adipate (*L_BAd_*) segments in the copolymers and the degree of randomness (*R*) according to Equations (3)–(5). The degree of randomness was slightly over 1 for all copolymers (Table 2), suggesting not only a completely random structure but even alternating sequences.

(3) $$L_{BF}=1+\frac{2\times I_{FBF}}{I_{FBAd}+I_{AdBF}},$$

(4) $$L_{BAd}=1+\frac{2\times I_{AdBAd}}{I_{FBAd}+I_{AdBF}},$$

(5) $$R=\frac{1}{L_{BF}}+\frac{1}{L_{BAd}},$$

where *I_BF_* is the integration of the butylene furanoate peak, *I_FBF_* is the integration of the peak of the FBF segments, *I_AdBAd_* is the integration of the peak of the AdBAd segments and *I_AdBF_* is the integration of the peak of the AdBF segments in the ^1^H NMR spectrum.

### 3.2. Characterization of the FDCA-Based Polyesters

Poly(butylene adipate-co-butylene 2,5-furandicarboxylate)s in several comonomer ratios were reported in the literature [6,10,13,14,32]. PBF-co-PBAd with low FDCA content (≤60 mol%) are degradable via hydrolysis and composting and are remarkably elastic. The DSC thermograms of PBF, PBAd and their copolymers are shown in Figure S4. PBAd is a fast-crystallizing polyester with a glass transition T_g_ = −57.3 °C, a double melting peak at 55 and 60 °C, and a melt crystallization T_c_ = 31.4 °C. PBF melts at 170 °C and has T_g_ = 37.7 °C and T_cc_ = 103.4 °C, in agreement with previous reports [33,34,35,36]. The copolymers PBF-PBAd 75 25 and PBF-PBAd 50 50 show weak cold crystallization and melting during heating after quenching (Figure S4d), and PBF-PBAd 25 75 does not crystallize during heating. The effect of the composition (calculated using NMR) on the thermal transitions of the copolymers is shown in Figure S5. As expected, when increasing the butylene adipate content, the T_m_, T_cc_ and T_g_ are reduced because the incorporation of the flexible BAd segments increases the chain mobility [6]. The presence of a single T_g_ for all copolymers complies with a random sequence structure, in agreement with the calculations from the NMR (Table 2).

The XRD patterns of PBF, PBAd and their copolymers are presented in Figure S6. PBF has a triclinic unit cell, and the diffraction peaks of the planes (010) and (100) appear at 17.5° and 24.5° [37]. PBAd has diffractions peaks at 2θ = 17.9° (002), 19.7°, 20.3°, 21.8° (110), 22.5° (020) and 24.2° (021) [38]. PBAd can crystallize in different crystal forms depending on the crystallization conditions, namely, α- and β-form crystals [39]. Herein, PBAd was melt-quenched at room temperature and shows peaks from both α- and β-form crystals. While the α-form has a monoclinic unit cell, the β-form has an orthorhombic one [39]. The copolymer PBF-PBAd 25 75 has diffraction peaks of both PBF and PBAd, indicating both units can crystallize. Increasing the BF content to 50 and 75% turns the copolymers amorphous, showing weak diffraction peaks of the PBF moiety; therefore, the crystallization of both comonomers is suppressed due to incompatibility of the crystal lattices of PBF and PBAd. This observation supports the findings of the DSC data.

### 3.3. Microstructural Features and Spectroscopic Analysis of the Blends

The miscibility of polymer blends is controlled by many factors: viscosity ratio, composition, shear forces, elasticity, the characteristics of the interface, molecular weight and crystallinity are among them. Blends of PLA/PBAd with PBAd content ≥30 wt% are immiscible, which was attributed to the crystallization of PBAd in the PLA matrix [40]. Blends of PLA/PBF prepared using melt blending were reported in the literature, with PBF content ranging from 5 to 75 wt% [16,22,23]. All of them were immiscible, as the typical sea-island morphology was observed with SEM.

**Figure 1:**
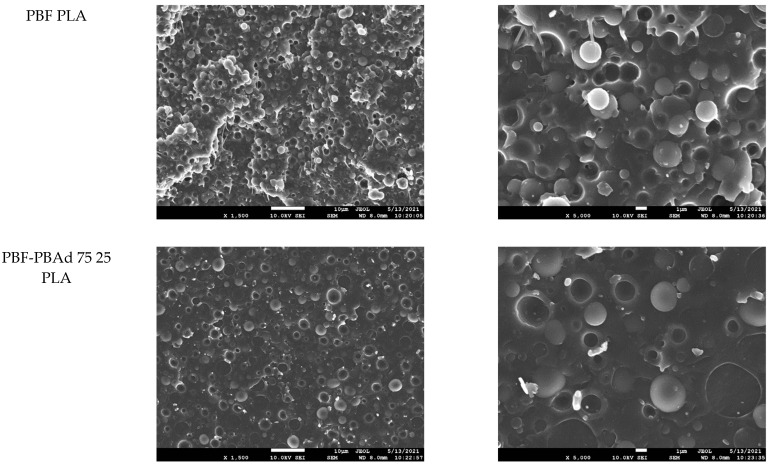

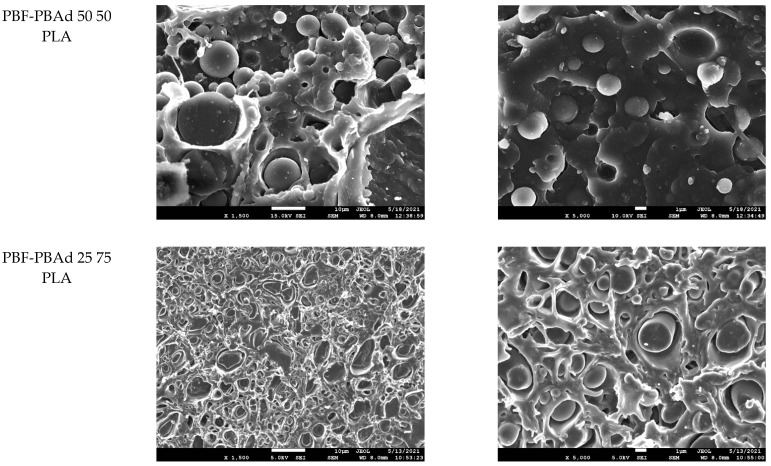
SEM micrographs of cryofractured cross-sections of the PLA-based blends. Left column magnification ×1000, right column magnification ×5000.

**Figure 2:**
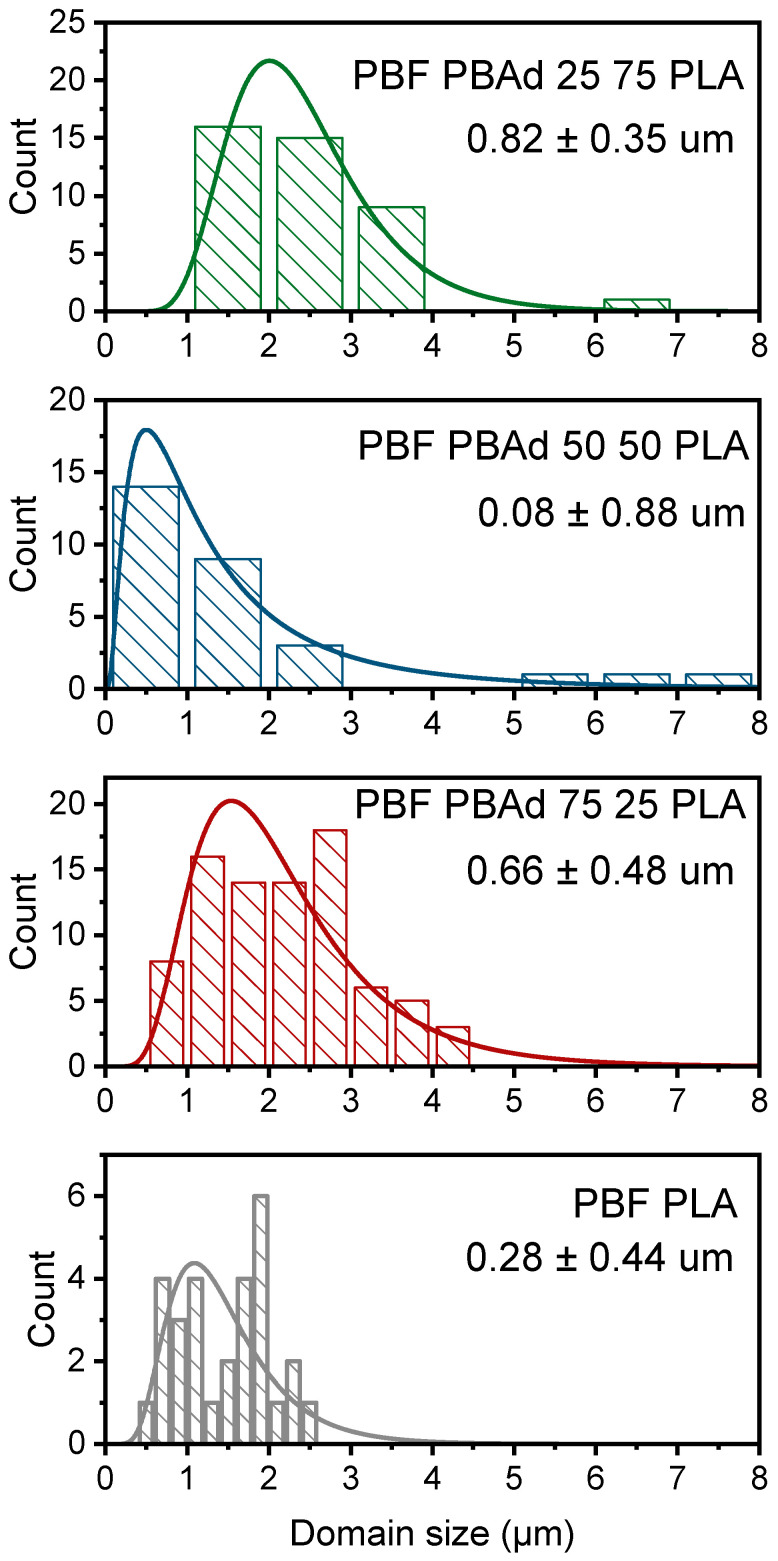
Histograms of the domain size and lognormal distribution curve.

The miscibility of the blends was first evaluated using SEM observations of cryofractured surfaces. The microphotographs are shown in Figure 1 at two different magnifications: ×1000 and ×5000. All blends show the sea-island morphology, which is typical for immiscible blends. The blends PBF PLA, PBF-PBAd 75 25 PLA and PBF-PBAd 50 50 PLA had small spherical particles of the second component with diameters from approximately 0.3 to 6 μm. On the blend PBF-PBAd 25 75 PLA, which contained the largest amount of PBAd among all the blends, the droplets had an irregular platelet-like shape rather than a spherical shape. All samples had empty cavities on their surfaces and evidence of debonding of the dispersed phase, but numerous domains remained attached, especially smaller ones, which can be attributed to transesterification reactions during melt blending [41]. The domain sizes were measured and fitted with a lognormal distribution. The lognormal distribution curves and the histograms, along with the lognormal mean domain sizes, are presented in Figure 2. The mean domain size varied between the blends; however, the standard deviation is quite large, suggesting that there were no significant variations between the samples. While there was a trend that showed an increase in the PBAd content in the dispersed phase also increased the mean domain size, the blend PBF-PBAd 50 50 PLA did not follow this trend, as a large number of smaller spherical droplets (≤0.5 μm) existed on its cryofractured surface. This smaller domain size hinted at slightly improved compatibility and could be attributed to the smaller intrinsic viscosity of the copolymer PBF-PBAd 50 50. The viscosity ratio of the dispersed phase to the matrix influences the blend miscibility and combining a high-viscosity matrix, such as PLA, with a low-viscosity dispersed phase yields a homogeneous and fine dispersion [42].

**Figure 3:**
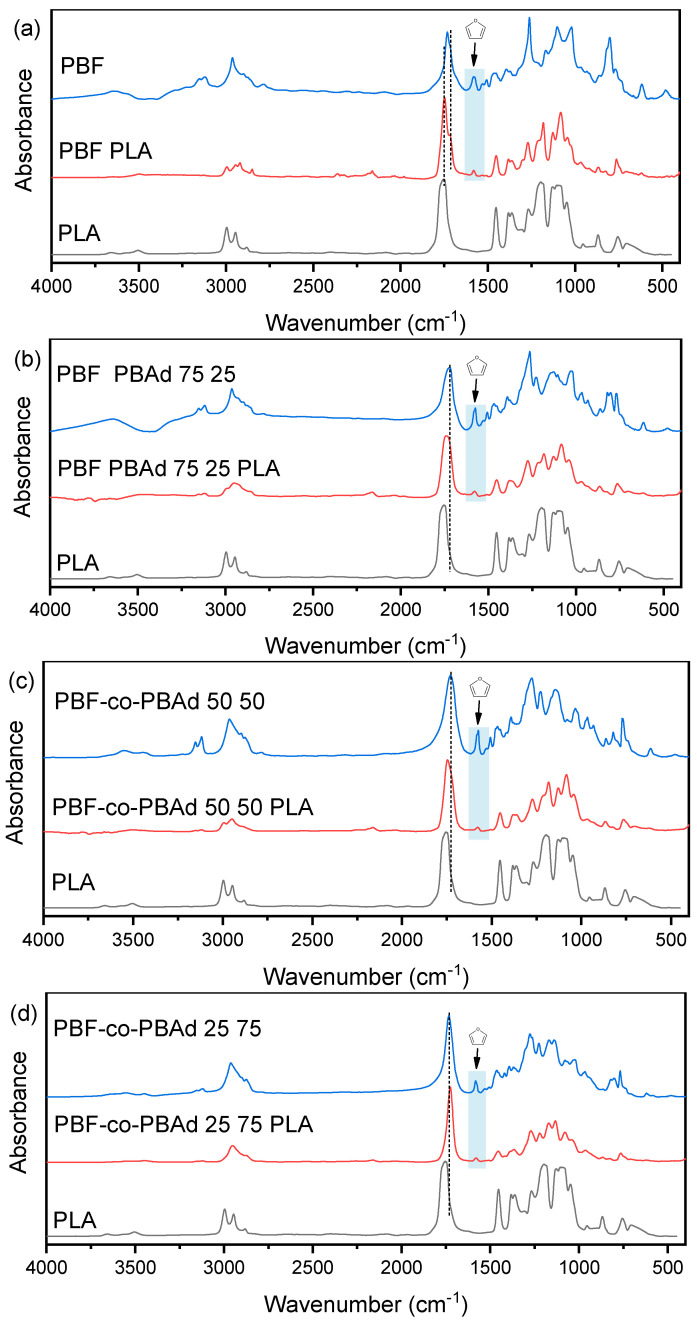
FTIR spectra of (**a**) PLA, PBF and PBF PLA blend (**b**) PLA, PBF-PBAd 75 25 and PBF-PBAd 75 25 PLA blend, (**c**) PLA, PBF-PBAd 50 50 and PBF-PBAd 50 50 PLA blend and (**d**) PLA, PBF-PBAd 25 75 and PBF-PBAd 25 75 PLA blend.

The FTIR spectra of the homopolymers, the copolymers and their blends are shown in Figure 3. PLA, due to its high molecular weight, had weak absorption bands of terminal hydroxyls at 3500 cm^−1^. The C-H stretching appeared at ~2900 cm^−1^ and ~2945 cm^−1^, the C=O stretching at 1752 cm^−1^, the -CH_3_ asymmetric stretching at 1453 cm^−1^ and the C-O-C stretching at 1185–1080 cm^−1^. The spectra of PBF and its blend with PLA are presented in Figure 3a. In the spectrum of PBF, the terminal -OH peak at ~3600 cm^−1^ was visibly stronger than in PLA. The asymmetric and symmetric stretching of the furan ring can be seen at 3150 cm^−1^ and 3120 cm^−1^, respectively [10,15,43]. The bands in the range of 2900–2800 cm^−1^ corresponded to the vibrations of the methyl groups of butanediol, and the band at 1732 cm^−1^ to the C=O stretching. The bands of the C-H and C=H vibrations of the furan ring were visible at 1580 and 1530 cm^−1^, respectively [10,36]. After blending PLA with the FDCA-based polyesters, a combination of the bands of the two components appeared on the spectra (Figure 3, middle spectra). It is noteworthy that the band of the terminal -OH groups was weaker, and the position of the C=O band of the FDCA-based component shifted to smaller wavenumbers, indicating that some limited transesterification reactions might have taken place during the melt blending. In contrast, no such reactions could be hypothesized when blends of PLA and FDCA-based polyesters were prepared via solvent casting [15]. The C=O absorption band did not shift in the spectrum of the blend PBF-PBAd 25 75 PLA (Figure 3d middle spectrum), which could have been due to the higher viscosity of the copolymer PBF-PBAd 25 75 in comparison with PBF-PBAd 50 50 and PBF-PBAd 75 25.

### 3.4. Thermal Properties and Crystallization of the Blends

The most common method used to assess whether two polymers are miscible is measuring the changes in their glass transition temperature (T_g_) via DSC. That is feasible only when the two T_g_ values differ significantly such that they can be detected. When a blend exhibits a single T_g_, miscibility is assumed, while two T_g_ values indicate partial miscibility or immiscibility [44].

**Figure 4:**
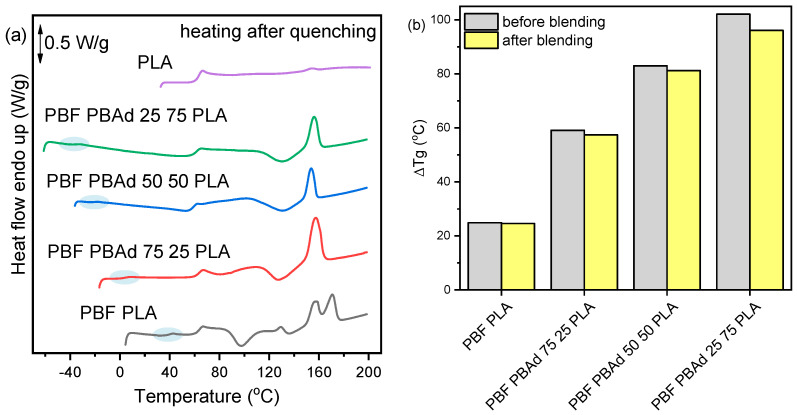
(**a**) DSC scans of the blends during heating after quenching at a rate of 20 °C/min and (**b**) ΔT_g_ values of the blend’s components before and after the melt blending.

The DSC traces of the prepared blends during heating after quenching are shown in Figure 4a. PLA has a T_g_ = 62.6 °C and very weak cold crystallization at T_cc_ = 126.5 °C. The formed crystals melted at T_m_ = 153.3 °C. The two components of the blends had significantly different T_g_ values, with ΔT_g_ ranging from ~25 to 100 °C (Figure 4b, gray columns). After mixing, the two T_g_ values were still present, confirming the immiscibility of the blends, and the ΔT_g_ reduced after blending. All blends crystallized during heating, and the crystallization of PLA in the blends was a lot more pronounced than in neat PLA as the ΔH_cc_ increased, suggesting the dispersed phases facilitated the cold crystallization of PLA. The blending of a polymer with an incompatible component can improve its nucleation rate, and therefore its crystallization [45]. This improvement in crystallization could be the result of heterogeneous nucleation occurring along the interface of the two phase-separated domains [46]. It was previously concluded that blending PBF with PLA led to increased PLA crystallinity [23]. Both PBF and PLA crystallized in the PBF PLA blend since two distinct cold crystallization peaks were detected at 97.4 °C and 136.2 °C, as well as two melting peaks at 170.8 °C and 157.5 °C corresponding to PBF and PLA, respectively. The T_cc_ and the T_m_ of PLA shifted to higher temperatures, but the T_cc_ of PBF shifted slightly toward lower temperatures in comparison with the neat polyesters. The blends with the PBF-PBAd copolymers did not show detectable cold crystallization of the dispersed phase, as the copolymers alone did not crystallize significantly.

**Figure 5:**
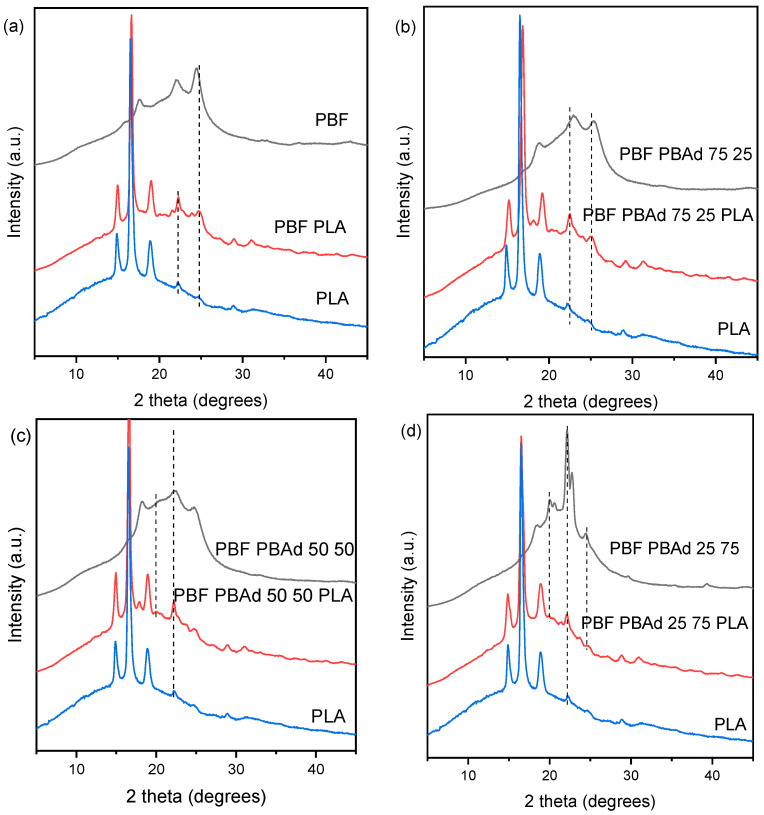
XRD patterns of isothermally cold-crystallized (**a**) PLA, PBF and PBF PLA blend (**b**) PLA, PBF-PBAd 75 25 and PBF-PBAd 75 25 PLA blend, (**c**) PLA, PBF-PBAd 50 50 and PBF-PBAd 50 50 PLA blend and (**d**) PLA, PBF-PBAd 25 75 and PBF-PBAd 25 75 PLA blend.

The XRD patterns of PLA, the FDCA-based polyesters and their blends are presented in Figure 5. The unit cell of PLA is orthorhombic, with the main diffraction peaks at 2θ = 15, 16.5 and 19°, which correspond to the (010), (200/110) and (203) crystal planes, respectively. The small peak at 22.3° is attributed to the (210) plane [47]. After annealing at the T_cc_ for 1 h, all the prepared blends showed the diffraction peaks of PLA, and some smaller peaks of the dispersed phases, suggesting that both components crystallized during isothermal cold crystallization (annealing). The peaks of the dispersed phase were clearly visible for the blends PBF PLA and PBF-PBAd 25 75, as those polymers had strong diffraction peaks. In the blends PBF-PBAd 75 25 and PBF-PBAd 50 50, no safe conclusions could be drawn, as the weak diffraction peaks of these copolymers might have overlapped with the weak diffraction peak of PLA at about 25°. In our previous study, both PLA and PBF were able to crystallize in a wide range of PLA PBF compositions [16].

### 3.5. Nanomechanical Properties

The nanomechanical properties of the copolymers and their blends with PLA were measured through nanoindentation testing. In Figure S7, the representative indentation load–depth curves are illustrated, along with the FEA force–depth data that fit the experimental nanoindentation loading curve.

**Figure 6:**
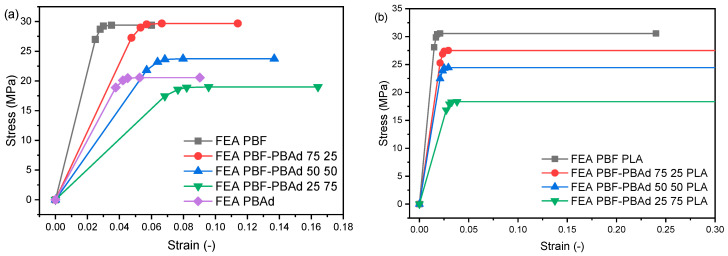
FEA generated stress–strain curves of (**a**) PBF, PBAd and PBF-co-PBAd, (**b**) PBF-PBAd PLA blends.

For the FE analysis, an initial value was introduced into the model for the first tangent modulus of the sample’s stress–strain curve. This value was related to the elastic modulus that was derived from the nanoindentation tests. The measured indentation depth was applied in steps to the FE model (on the indenter), and then the force reaction was computed and compared with the measured value. The FEA force–depth data should fit the experimental nanoindentation curve; else, the value of the tangent modulus has to be computed again. For the cases where the solutions returned a computational force matching the measured force, the value of the tangent modulus was considered to be accepted, and the next couple of values of force and depth were applied to the model. The following calculation steps started with the previous indentation depth value, considering the already existing stress status and the previously obtained tangent modulus. This process was repeated until the last couple of load–depth values converged and the loop ended. At least 20 simulation steps were considered sufficient to achieve converged FEA solutions and proceed to a satisfactory curve fitting of the nanoindentation curves. The potential to calculate the stress–strain curves of polymers based on the force–depth nanoindentation testing under varied conditions allowed for the estimation of the materials’ constitutive laws. The computationally generated stress–strain curves are presented in Figure 6. The resulting elastic modulus, ultimate stress and strain from the FEA-assisted nanoindentation testing (analytical–experimental method), as well as the elastic modulus and hardness from nanoindentation testing, are summarized in Table 3.

The results showed a good correlation between the measured nanoindentation tests and the computational data for all specimens that contained PLA (Figure 6b). For the PBF-PBAd copolymer specimens (Figure 6a), the FEA seemed to overpredict the elastic modulus results. This was due to the inherent nature of calculation from the Oliver–Pharr approach [26], which measures the elastic modulus from the unloading section of the load–depth curve, while the FEA approach calculates the response from the loading curve up to the maximum force. Overall, the results showed a significant increase in strength for the PLA-containing blends. Considering these results, it can be concluded that the PBF-PBAd copolymers affected the specimen’s overall stress–strain behavior by reducing the elastic modulus values; however, from the elasto-plastic type stress–strain curves, the ultimate strain increased due to the flexible macromolecular chain of PBAd.

Furthermore, the experimental nanoindentation technique assisted by FEA was shown to be a very successful method for determining the mechanical behavior of the materials. The values of elastic modulus obtained from the nanoindentation testing and FEA of the blends were almost similar and always in the same order of magnitude. A decreasing trend in the elastic modulus values of PBF-PBAd was observed with the increase of PBAd content in the copolymers, which was expected due to the flexible behavior of PBAd. A similar drop is observed in the values of hardness.

The elastic properties of the PLA matrix differed significantly from those of the dispersed phases since the PBF-PBAd copolymers had a much smaller elastic modulus when compared with PLA, which is a stiff polymer with small elongation. Thus, the blends had intermediate elastic modulus and stress at break values that decreased as the softness of the dispersed phase increased. Similar behavior was observed when PBAT was added in the PLA for PBAT content up to 40 wt% [48]. It was reported that when using only a small amount of PBF in the preparation of immiscible blends of PLA/PBF, the elongation and impact toughness of PLA significantly increased [22]. Herein, the amount of PBF used might have been too large to obtain a similar improvement in elongation. Overall, the materials PBF-PBAd 75 25 and PBF-PBAd 75 25 PLA presented the best nanomechanical properties during nanoindentation testing in comparison with all the PBF-PBAd copolymers and blends. However, it should be noted that since the blends are all immiscible, the cavities from the dispersed phase particles are expected to act as stress concentration points upon mechanical loading.

### 3.6. Wettability and Soil Burial Test

**Figure 7:**
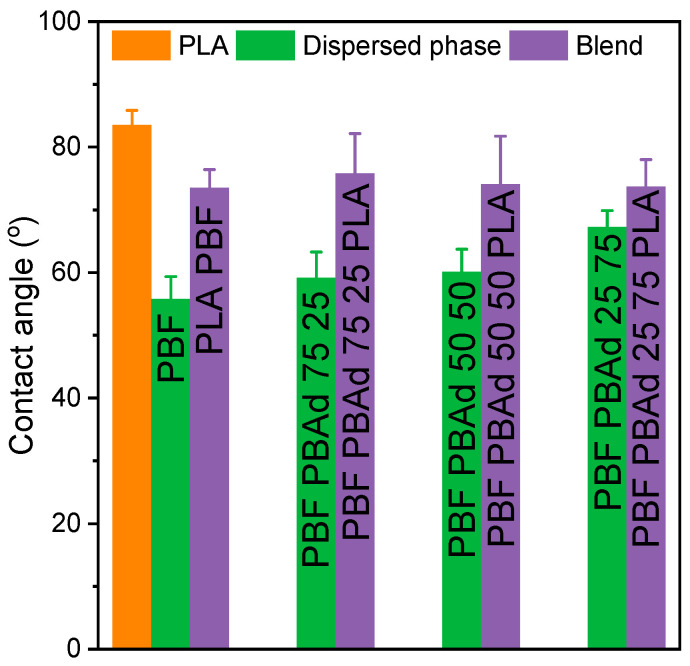
Water contact angle of the polymers before and after blending.

The effect of blending on the wettability of PLA was investigated by measuring the water contact angle. The values obtained are shown in Figure 7. Water contact angle values are related both to the composition of the material, as well as the roughness of the substrate [49]. The contact angle values of all the materials were <90°, which is considered hydrophilic and was attributed to the numerous oxygen species of the repeating units of the polyesters. The contact angle of PBF and its copolymers with PBAd are represented by the green bars of Figure 7. PBF has a contact angle of 55.8 ± 3.5°, and it was increased after copolymerization with PBAd, as well as with increased PBAd content. This increase was due to the introduction of the eight methylene groups of the PBAd repeating unit into the macromolecular chain. The contact angle of PBF reported in the literature was larger than in this study [43,50], which was likely a result of the higher molecular weight and, as a result, fewer polar terminal -OH and -COOH groups. PLA was the least hydrophilic of all the polymers in this work, as it had the highest molecular weight. After introducing the FDCA-based polymers in the PLA matrix, its contact angle decreased and, therefore, its hydrophilicity increased, and all the blends had contact angle values in between those of their components.

PLA degrades under industrial composting conditions (58 °C, 90% biodegradation up to 6 months) and is likely to degrade in thermophilic anaerobic digestion at 52 °C. PBAT is also degradable under industrial composting conditions and certain grades are susceptible to home composting and soil degradation [51]. PBF does not degrade after 8 weeks of composting [52]. PBF-co-PBAd with 40–60% BF was found to be compostable under standard conditions regulated in ISO 14855-1:2005 and GB/T 19277.2-2013, as they reached 90% biodegradation in 110 days [32]. As expected, increasing the BF content decelerated the biodegradation.

**Figure 8:**
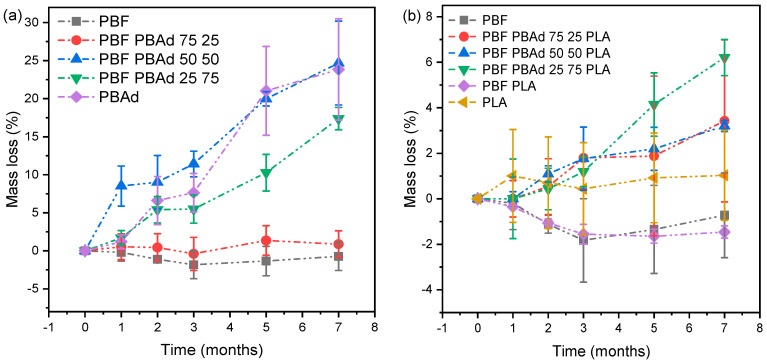
Cumulative mass loss of (**a**) PBF, PBAd and their copolymers, and (**b**) PBF, PLA and the blends during soil burial.

The hydrolysis rate of aliphatic–aromatic copolyesters depends on the sequence length of the aromatic unit, the hydrophilicity and the inherent sensitivity of the ester bonds to hydrolytic attack. Ester bonds adjacent to aliphatic moieties are more prone to hydrolysis [53,54]. In addition, in soil degradation, the soil type and quality affect degradation rates [55]. In the scope of this work, a preliminary evaluation of the degradability of the polymers in real environmental conditions was performed. The cumulative mass loss of the polymers studied is shown in Figure 8. As seen in Figure 8a, both PBF and PBF-PBAd 75 25 did not lose significant mass after 7 months of burying. Increasing the BAd content to 50 and 75 mol% enabled measurable mass loss, which reached ~25% in 7 months. In fact, PBF-PBAd 50 50 degraded similarly to PBAd, which contained only aliphatic units in its macromolecular chain. The accelerated degradation of this copolymer can be attributed to its lower molecular weight. PLA and its blends (Figure 8a) showed a different mass loss pattern since PLA and the matrix of the blends were not degradable in soil. Both PBF and the blend PBF PLA seemed to gain weight, which was attributed to the contamination of their surface with soil and a simultaneous lack of mass loss. PLA showed an insignificant mass loss with a large standard deviation; therefore, it was considered completely non-degradable under the testing conditions. After blending it with the FDCA-based copolyesters, a small mass loss was observed, reaching ~6% for the blend PBF-PBAd 25 75 PLA. This mass loss might be limited but it is an indication that having only a small fraction of the biodegradable repeating BAd units in the blend composition could accelerate the degradation of PLA. The properties that dictate degradation rates of the materials are molecular weight and hydrophilicity. Overall, for the samples that degraded, mass loss was increasing faster after 3 months of burying, which could be associated with the higher temperatures in that timeframe (Figure S1).

**Figure 9:**
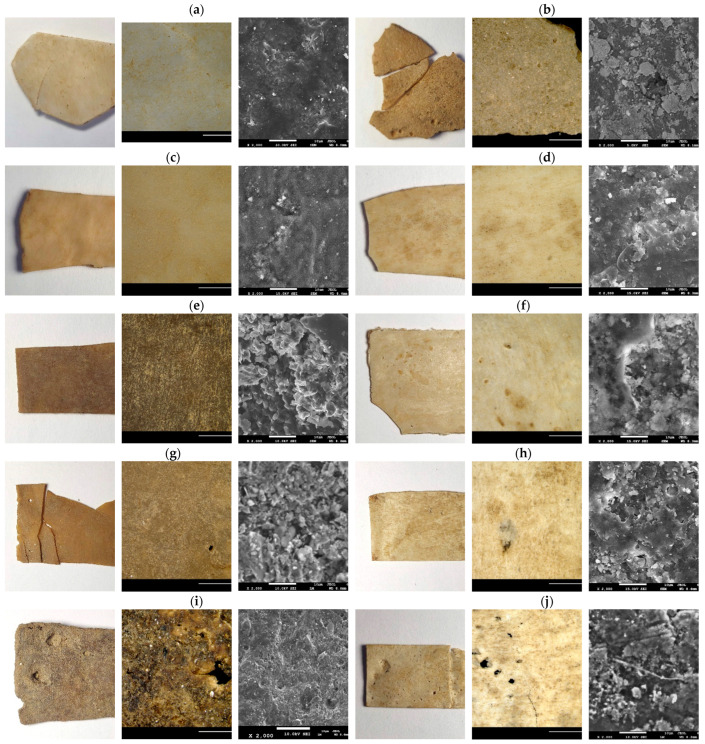
The surfaces of the materials after 7 months of soil degradation. From left to right: macroscopic photograph, stereoscope image with ×7.5 magnification and SEM image with ×2000 magnification. Reactive blending. (**a**) PLA; (**b**) PBAd; (**c**) PBF; (**d**) PBF PLA; (**e**) PBF-PBAd 75 25; (**f**) PBF-PBAd 75 25 PLA; (**g**) PBF-PBAd 50 50; (**h**) PBF-PBAd 50 50 PLA; (**i**) PBF-PBAd 25 75; (**j**) PBF-PBAd 25 75 PLA.

The appearance of the samples after being buried for 7 months is shown in Figure 9. PLA was not significantly affected macroscopically, except for the contamination of its surface by soil residues (Figure 9a). PBAd broke into several fragments and its color turned from white to brown (Figure 9b). Among the PBF-PBAd copolymers, macroscopic changes were noticed for PBF-PBAd 50 50 (Figure 9g) and PBF-PBAd 25 75 (Figure 9i), which had the lowest molecular weights and the highest BAd contents. These two films had holes visible to the naked eye and started breaking after 5 months of burying. The appearance of the blends was affected the most in terms of coloring with contamination from the soil, and some holes started appearing at the thinnest parts of the films of the blend PBF-PBAd 25 75 PLA, Figure 9j, which agreed with the mass loss results and revealed the important role of film thickness on degradation rates. The microscopic changes in the surface of the materials were examined with SEM. Besides PLA and PBF (Figure 9a,c), all polymers had very rough surfaces with defects and holes, suggesting surface erosion, as well as deposited particles. These could be traces of soil, mineral deposits or biofilms. In Figure 9j, a fiber-like structure that resembled microbial biofilms formed on the PBF-PBAd 25 75 PLA blend.

### 3.7. Reactive Blending

Reactive blending was performed to examine whether it can improve the compatibility between the components of the blends. In general, reactive blending concerns the mixing of the blend at elevated temperatures for prolonged times to enable transesterification reactions to occur. Increasing the blending time leads to the formation of block copolymers initially, and further increasing it yields random copolymers. Reactive blending was simulated inside the DSC pan to make a preliminary estimation of its effect on miscibility improvement. To do so, the blends were held isothermally at 220 °C or 250 °C for different times ranging from 10 to 50 min. After each isothermal step, a quenching and a heating scan with a heating rate of 20 °C/min were performed to measure the T_g_. The start of thermal degradation during reactive blending was concluded from the DSC curves of the isothermal steps.

**Figure 10:**
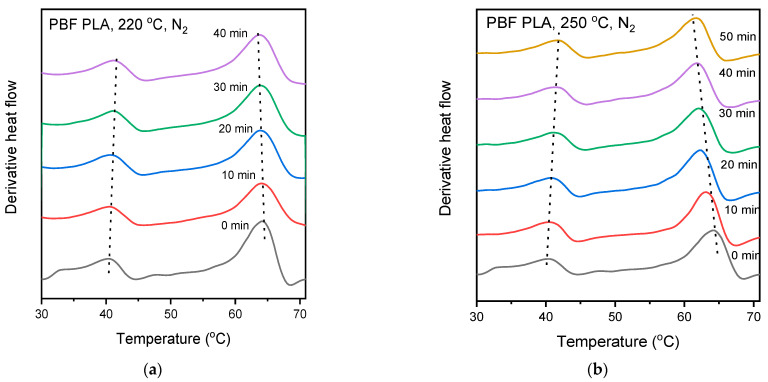

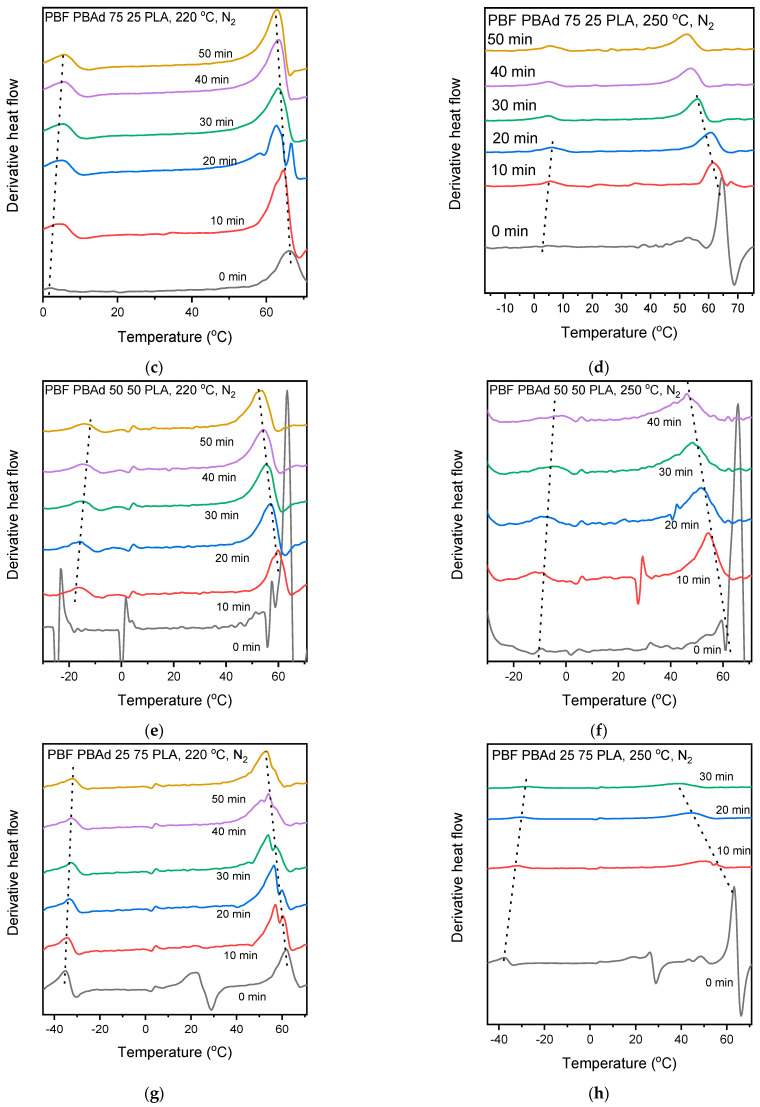
Study of reactive blending with DSC: derivative heat flow and change in the T_g_ of the (**a**,**b**) PBF PLA, (**c**,**d**) PBF-PBAd 75 25 PLA, (**e**,**f**) PBF-PBAd 50 50 PLA and (**g**,**h**) PBF-PBAd 25 75 PLA blends.

The effect of the reactive blending time on the T_g_ of the blends is shown in Figure 10. In general, when T_g_ values gradually approach each other, it indicates compatibilization [56]. Another indication for improved miscibility is the suppression of cold crystallization and a decrease in the T_m_ since the ability of copolymers to crystallize is limited in comparison with blends.

In the PBF PLA blend, only the T_g_ of PBF increased during reactive blending at 220 °C (Figure 10a); the cold crystallization (Figure S9a) shifted slightly toward higher temperatures, indicating reduced macromolecular mobility; and the positions of the T_m_ peaks were not significantly affected. The total melting enthalpy ΔH_m_ reduced with time, which was reflected by the smaller area of the melting peaks, showing the reduced crystallinity after reactive blending. At 250 °C, the T_g_ of both PLA and PBF shifted toward each other (Figure 10b), cold crystallization was suppressed significantly, the T_m_ values shifted to lower temperatures and the two distinct melting peaks merged into one ((Figure S9c). The reduction in the T_m_ could be a result of less perfected crystallites due to reduced chain mobility as transesterification reactions take place. TGA measurements of the PBF PLA blend did not show any significant mass loss up to 300 °C (Figure S8).

The change in the T_g_ of the blend PBF-PBAd 75 25 PLA during the reactive blending is shown in Figure 10c,d. At 220 °C, the two T_g_ values approached each other up to 20 min, and after that, their changes remained insignificant. Signs of degradation started showing up on the DSC curves after 40 min of reactive blending as a small exothermic decline of the baseline (data not shown). The T_m_ and the T_cc_ were reduced with blending time, and the extent of cold crystallization remained similar. At 250 °C, reactive blending seemed to be efficient again until 20 min, with reduced ΔT_g_ and T_m_ values, as well as suppressed cold crystallization, but thermal degradation occurred for prolonged times. Indeed, the TGA curves (Figure S8) showed that the blend PBF-PBAd 75 25 PLA lost 1.5% at 220 °C and ~5% of its initial mass at 250 °C.

The PBF-PBAd 50 50 PLA blend was thermally stable up to 275 °C (Figure S8); therefore, extensive degradation was not expected to occur during reactive blending. At 220 °C and with 40 min of reactive blending, the T_g_ of PLA reduced by 9.2 °C, and the T_g_ of the dispersed phase increased by 7 °C (Figure 10e). Based on the shift of the T_g_ values, transesterification reactions might have occurred for the first 30 min. After that, the changes in both T_g_ were subtle. The T_cc_ and T_m_ of PLA shifted to lower temperatures with time, but the overall crystallinity was sustained after reactive blending. As the T_cc_ of PLA becomes smaller, the T_m_ splits into two peaks because PLA crystallizes into both α and α’ form crystals when the T_cc_ decreases [57]. Despite the good thermal stability of the blend PBF-PBAd 50 50 PLA, thermal degradation was detected using DSC after the reactive blending at 250 °C for times ≥20 min. After 20 min, the shift in the T_g_ values (Figure 10f) became less pronounced, marking the competition between potential transesterification and degradation reactions. The T_cc_ increased and crystallization of PLA was suppressed, which are additional indications that transesterification reactions might have occurred during the reactive blending for 10 and 20 min at 250 °C.

The blend PBF-PBAd 25 75 PLA was thermally stable at 220 °C in TGA and started losing mass right after 250 °C (Figure S8). During reactive blending at 220 °C (Figure 10g), the T_g_ of the blend shifted toward each other and, like the rest of the blends, the shift was more intense during the first 20 min. The T_cc_ increased and the extent of the crystallization remained unaffected. At 250 °C (Figure 10h), degradation had occurred already during the first 10 min; therefore, reactive blending of the blend PBF-PBAd 25 75 PLA was not possible at this temperature.

The largest shift in T_g_ values after 20 min of reactive blending occurred for the blend PBF-PBAd 50 50 PLA, both at 220 °C and 250 °C. Thus, reactive blending had the most prominent effect on the improvement of the compatibility of this specific blend. This observation was in line with the observed smaller domain size of PBF-PBAd 50 50 PLA, which was correlated to the smaller viscosity of the dispersed phase PBF-PBAd 50 50.

**Figure 11:**
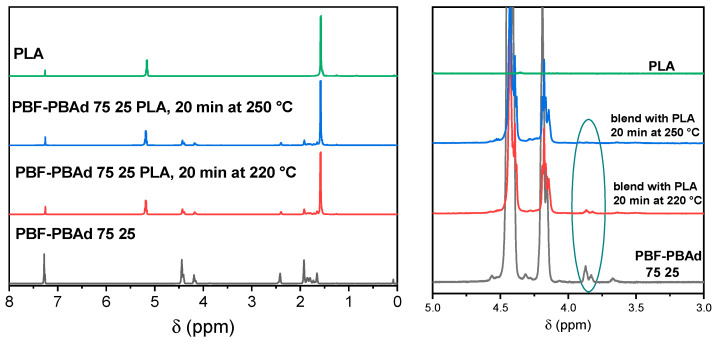
^1^H NMR spectra of the PBF-PBAd 75 25 PLA blends.

To further investigate the effect of reactive blending on the chemical structure of the blends, NMR spectra were recorded. Indicative NMR spectra of the PBF-PBAd 75-25 PLA blends obtained after 20 min at 220 °C and 250 °C are displayed in Figure 11, while the ^1^H NMR spectra of the other blends are presented in Figure S13. Resonance signals attributed to PLA were observed at 5.19 ppm (OCH) and 1.59 ppm (CH_3_), in addition to the PBF-PBAd copolymer peaks (vide supra). New peaks indicative of the formation of bonds between PLA and PBF-PBAd copolymers could not be observed. Nevertheless, the −CH_2_OH end groups of PBF-PBAd copolymers decreased during the blending, strongly suggesting some transesterification reactions occurred with PLA. Furthermore, this decrease was proportional to the temperature, confirming a more effective blending at 250 °C. The blends composition was calculated using the integrals of the peaks at 5.19 ppm and 4.43 ppm for PBF, or 4.14–4.49 and 4.38–4.43 ppm for PBF-PBAd copolymers and was found in accordance with the feed ratio.

## 4. Conclusions

PLA/PBF and PLA/PBF-co-PBAd blends with 70 wt% PLA were prepared using melt blending and their physicochemical properties were evaluated. The blends were immiscible (confirmed using SEM and DSC) and the domain size had an increasing trend with increasing PBAd content, except for the blend PBF-PBAd 50 50 PLA, which had the smallest domain size due to the smaller intrinsic viscosity of the copolymer PBF-PBAd 50 50. During the melt blending, some transesterification reactions might have occurred, as detected in the FTIR spectra. In all blends, the PLA matrix was able to crystallize during annealing. Nanomechanical characterization revealed that the elastic modulus, stress at break and hardness of the PLA reduced after the addition of the dispersed phases. FEA allowed for the generation of stress–strain curves that showed a good correlation between the measured nanoindentation tests and the computational data and confirmed the improvement in the case of the presence of PLA. Soil degradation was studied using mass loss quantification and surface observation with microscopy. The blends showed a small but detectable mass loss of the blends, which was accompanied by contaminated surfaces and microscopic holes, among other defects. Finally, reactive blending was found to be a possible compatibilizer-free approach to improve blend miscibility since it reduced the ΔΤ_g_ of the blend’s components.

## Figures and Tables

**Table 1:** Intrinsic viscosity values of the synthesized oligomers and polymers.

Sample	[*η*] (dL/g)	Mn (g/mol)	PDI
PBAd oligo	0.26	3600	2.18
PBAd	0.47	15,400	2.28
PBF oligo	0.06	N.D.	N.D.
PBF	0.6	N.D.	N.D.
PBF-PBAd 75 25	0.54	23,600	2.59
PBF-PBAd 50 50	0.41	9900	2.25
PBF-PBAd 25 75	0.63	15,790	2.25

N.D.: not determined.

**Table 2:** Comonomer ratio in the feed calculated with NMR, as well as the block length and degree of randomness of the PBF-co-PBAd copolyesters.

PBF-PBAd Feed Ratio (mol%)	Ratio Calculated with NMR (mol%)	*L_BF_*	*L_BAd_*	*R*
PBF-PBAd 75 25	74–26	2.0	1.1	1.41
PBF-PBAd 50 50	60–40	1.7	1.4	1.31
PBF-PBAd 25 75	31–69	1.4	3.7	1.00

**Table 3:** Mechanical properties of the homopolymers, copolymers and blends obtained using nanoindentation and FEA-assisted nanoindentation.

Property	Elastic Modulus	FEA Elastic Modulus	FEA Ultimate Stress	Nanoindentation Hardness
Units	MPa	MPa	MPa	MPa
PLA	3572 ± 260	3600	60	142 ± 7.4
PBF	774 ± 43	1080	N.D.	39.2 ± 3.1
PBF PLA	2260 ± 107	1873	30.569	125.5 ± 11.4
PBF-PBAd 75 25	217.7 ± 4.51	574	29.679	14.5 ± 0.7
PBF-PBAd 75 25 PLA	1262 ± 38	1204	27.512	72.9 ± 0.4
PBF-PBAd 50 50	35 ± 1	383	23.743	2.6 ± 0.08
PBF-PBAd 50 50 PLA	1004 ± 16.4	1070	24.455	66.7 ± 2.8
PBF-PBAd 25 75	31 ± 0.43	255	18.994	1.9 ± 0.08
PBF-PBAd 25 75 PLA	561 ± 19.3	617	18.342	37.7 ± 1.5
PBAd	506.03 ± 57	502	20.570	29.72 ± 5.5

N.D.: not determined.

## Data Availability

Data supporting reported results can be found within the article and the Supplementary Materials.

## Supplementary Materials

The following supporting information can be downloaded from https://www.mdpi.com/article/10.3390/polym14214725/s1. Figure S1: Average daily temperature in Thessaloniki, Greece, during the soil degradation testing. Figure S2: NMR spectra of the synthesized copolymers. (a) ^1^H NMR, (b) ^13^C NMR, (c) ^1^H NMR zoom in the 1.5–4.5 ppm region and (d) numbered structures of PBF-PBAd copolymers. The peaks with the asterisk are assigned to the deuterated solvent (CDCl_3_ and TFA-d1). Figure S3: Possible triads of PBF-PBAd copolymers. Figure S4: DSC scans of the homopolymers and copolymers during (a) 1st heating with a rate of 20 °C/min, (b) 2nd heating with a rate of 20 °C/min, (c) cooling with a rate of 10 °C/min and (d) heating after quenching with a rate of 20 °C/min. Figure S5: Thermal characteristics of the homopolymers PBF, PBAd and their copolymers. Figure S6: XRD patterns of PBF, PBAd and their copolymers. Figure S7: Load–depth nanoindentation curves of the PBF, PLA, PBF-PBAd and PBF-PBAd PLA specimens, along with the curve-fitted FEA response. Figure S8: (a) TGA and (b) DTG curves of the PLA-based blends. Figure S9: Study of reactive blending with DSC: heating curves after different times, with a zoomed-in view of the T_g_ region of PBF PLA at 220 °C (a,b) and (c,d) 250 °C. Figure S10: Study of reactive blending with DSC: heating curves after different times, with a zoomed-in view of the Tg region of PBF-PBAd 75 25 PLA at 220 °C (a,b) and (c,d) 250 °C. Figure S11: Study of reactive blending with DSC: heating curves after different times, with a zoomed-in view of the Tg region of PBF-PBAd 50 50 PLA at 220 °C (a,b) and (c,d) 250 °C. Figure S12: Study of reactive blending with DSC: heating curves after different times, with a zoomed-in view of the T_g_ region of PBF-PBAd 25 75 PLA at 220 °C (a,b) and (c,d) 250 °C. Figure S13: ^1^H NMR spectra of (a) PBF PLA, (b) PBF-PBAd 50-50 PLA, (c) PBF-PBAd 25-75 PLA blends, at 220 °C and 250 °C.
