# Supplementary material for: Blending PLA with Polyesters Based on 2,5-Furan Dicarboxylic Acid: Evaluation of Physicochemical and Nanomechanical Properties
Source: Polymers (Basel). 2022 Nov 4;14(21):4725. doi: 10.3390/polym14214725 (PMC9654909; doi:10.3390/polym14214725)
Supplement: Supplementary file 1 [file polymers-14-04725-s001.zip › polymers-2009416-supplementary.pdf]

Supplementary Materials

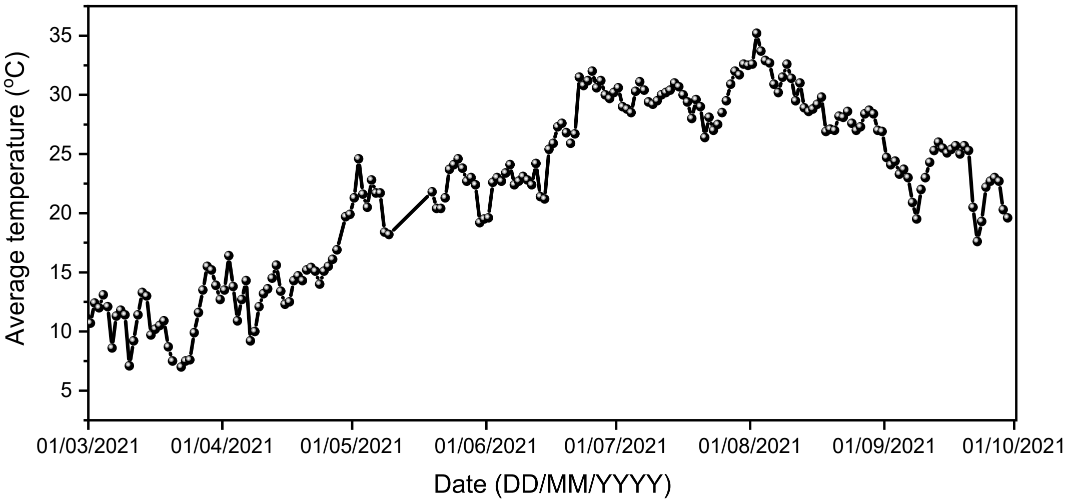

Figure S1. Average daily temperature in Thessaloniki, Greece, during soil degradation testing.

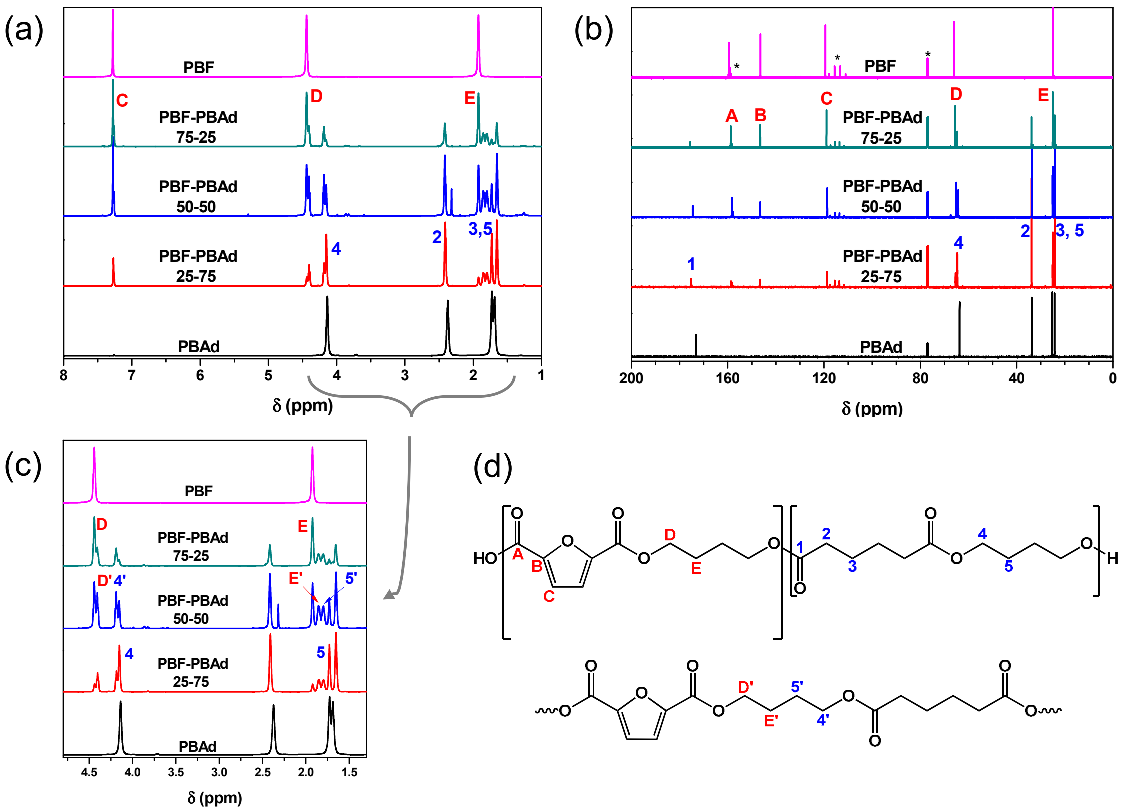

Figure S2. NMR spectra of the synthesized copolymers. (a) ^{1}H NMR, (b) ^{13}C NMR, (c) ^{1}H NMR zoom in the 1.5 - 4.5 ppm region, (d) numbered structures of PBF-PBAd copolymers. The peaks with the asterisk are assigned to the deuterated solvent ($CDCl_3$ and TFA-d_1).

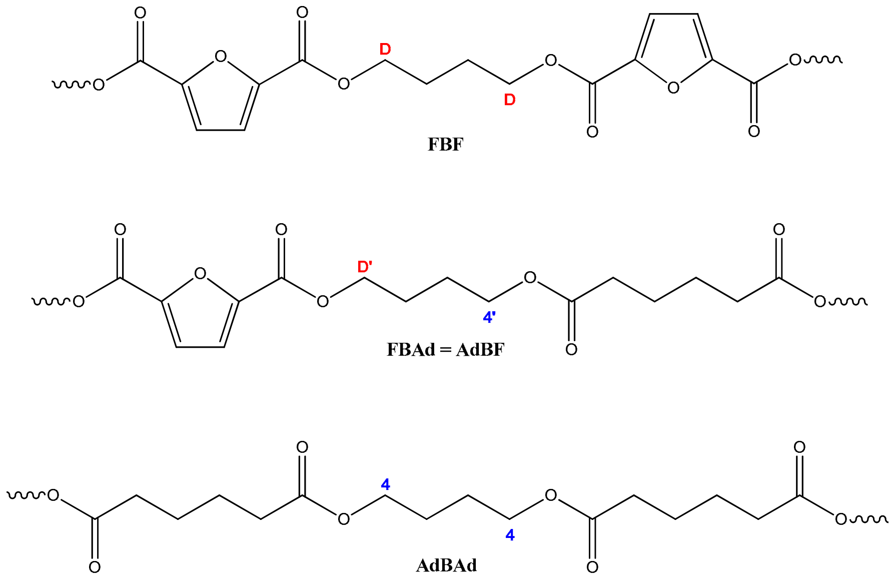

Figure S3. Possible triads of PBF-PBAd copolymers.

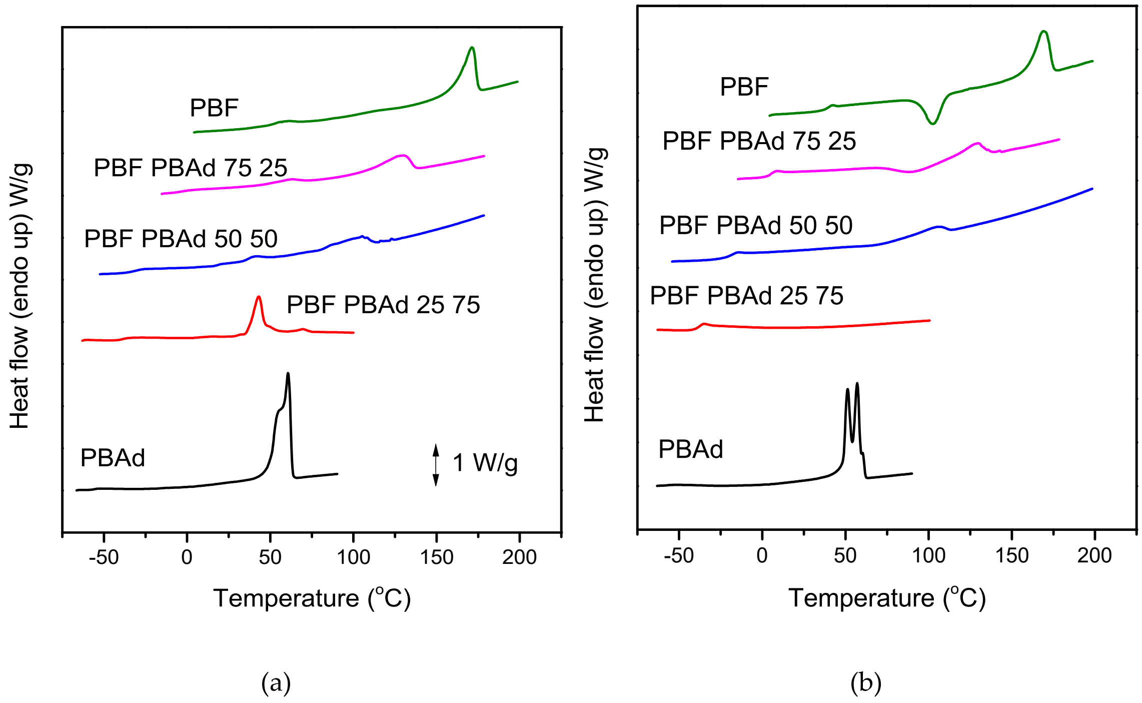

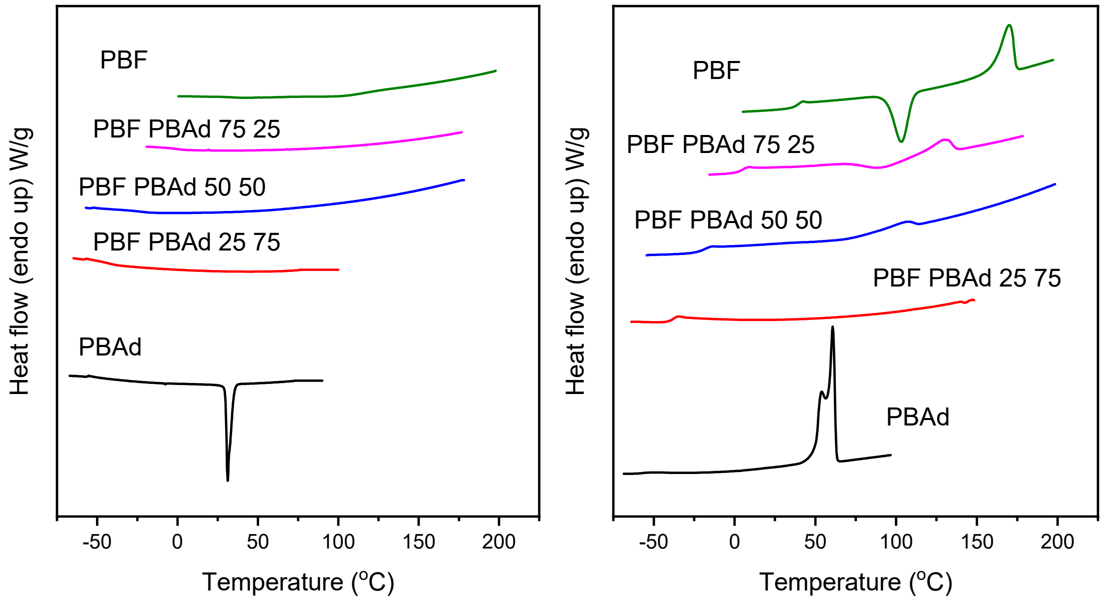

(c) (d)

Figure S4. DSC scans of the homopolymers and copolymers during (a) 1st heating with rate 20 ºC/min, (b) 2nd heating with rate 20 ºC/min, (c) cooling with rate 10 ºC/min and (d) heating after quenching with rate 20 ºC/min.

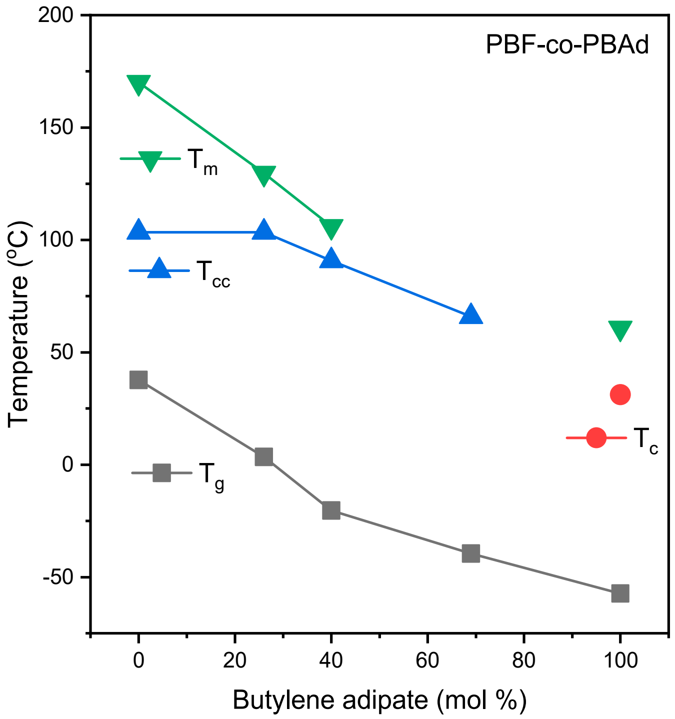

Figure S5. Thermal characteristics of the homopolymers PBF, PBAd and their copolymers.

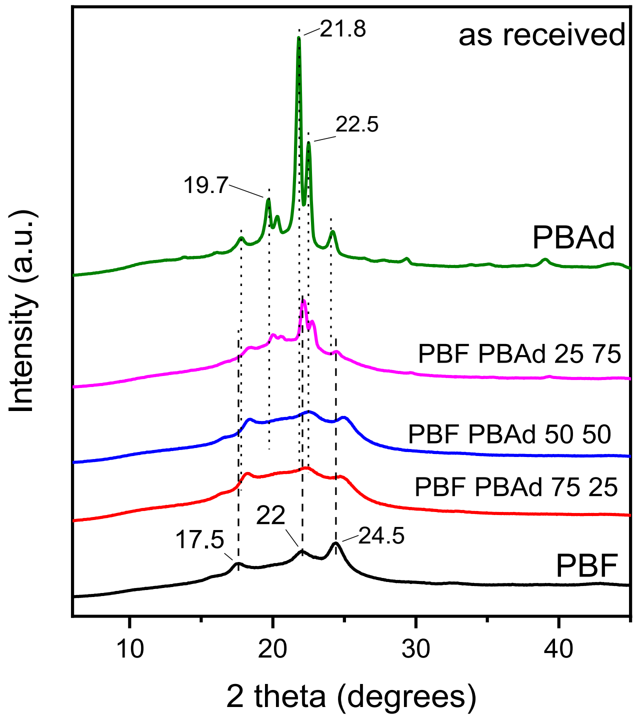

Figure S6. XRD patterns of PBF, PBAd and their copolymers.

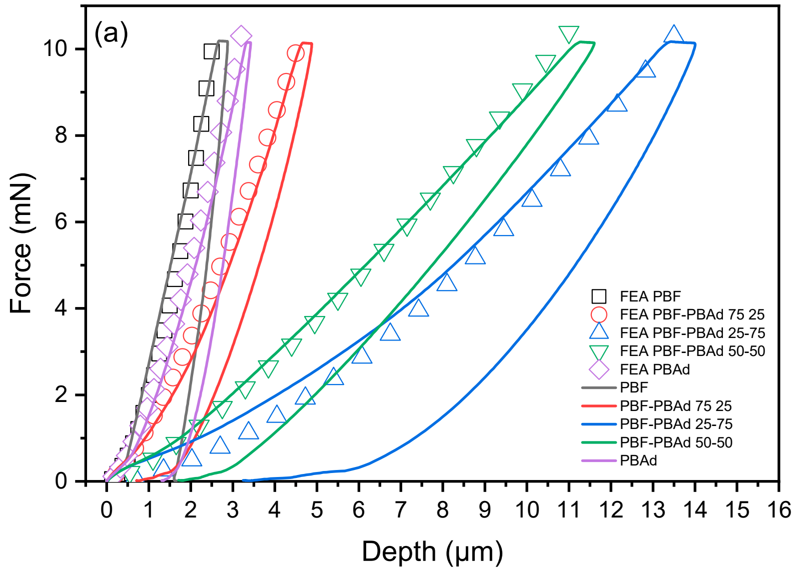

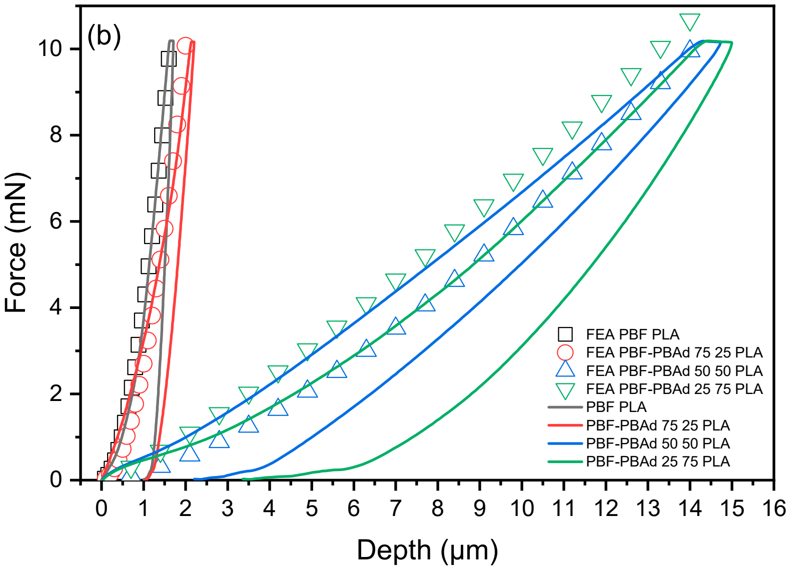

Figure S7. Load–depth nanoindentation curves of the PBF, PLA, PBF-PBAd and PBF-PBAd PLA specimens along with the curve-fitted FEA response.

Table S1. Thermal characteristics of PLA and its blends during heating after quenching, measured by DSC.

Sample	T_g	T_m	ΔH_m	T_{cc}	DH_{cc}
	°C	°C	J/g	J/g	J/g
PBF PLA	38.2, 62.8	157.5, 170.8	16.6	97.4, 136.2	-9.5
PBF-PBAd 75 25 PLA	4.2, 61.6	157.5	11.2	127.5	-10
PBF-PBAd 50 50 PLA	-22.6, 58.6	153.8	7.1	131.1	-11.3
PBF-PBAd 25 75 PLA	-35.6, 60.5	167.3	9.6	131.0	-9.0
PLA	62.6	153.3	0.5	126.5	-0.5

T_g: glass transition temperature, T_m: melting point, ΔH_m: melting enthalpy, T_{cc}: cold crystallization temperature, ΔH_{cc}: cold crystallization enthalpy, T_c: melt crystallization temperature, ΔH_c: melt crystallization enthalpy.

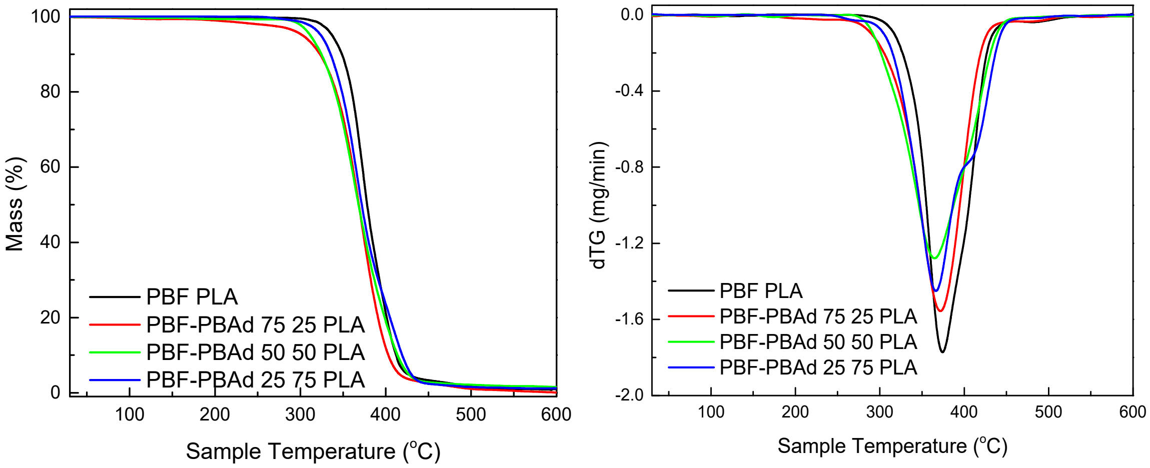

Figure S8. (a) TGA and (b) DTG curves of the PLA-based blends.

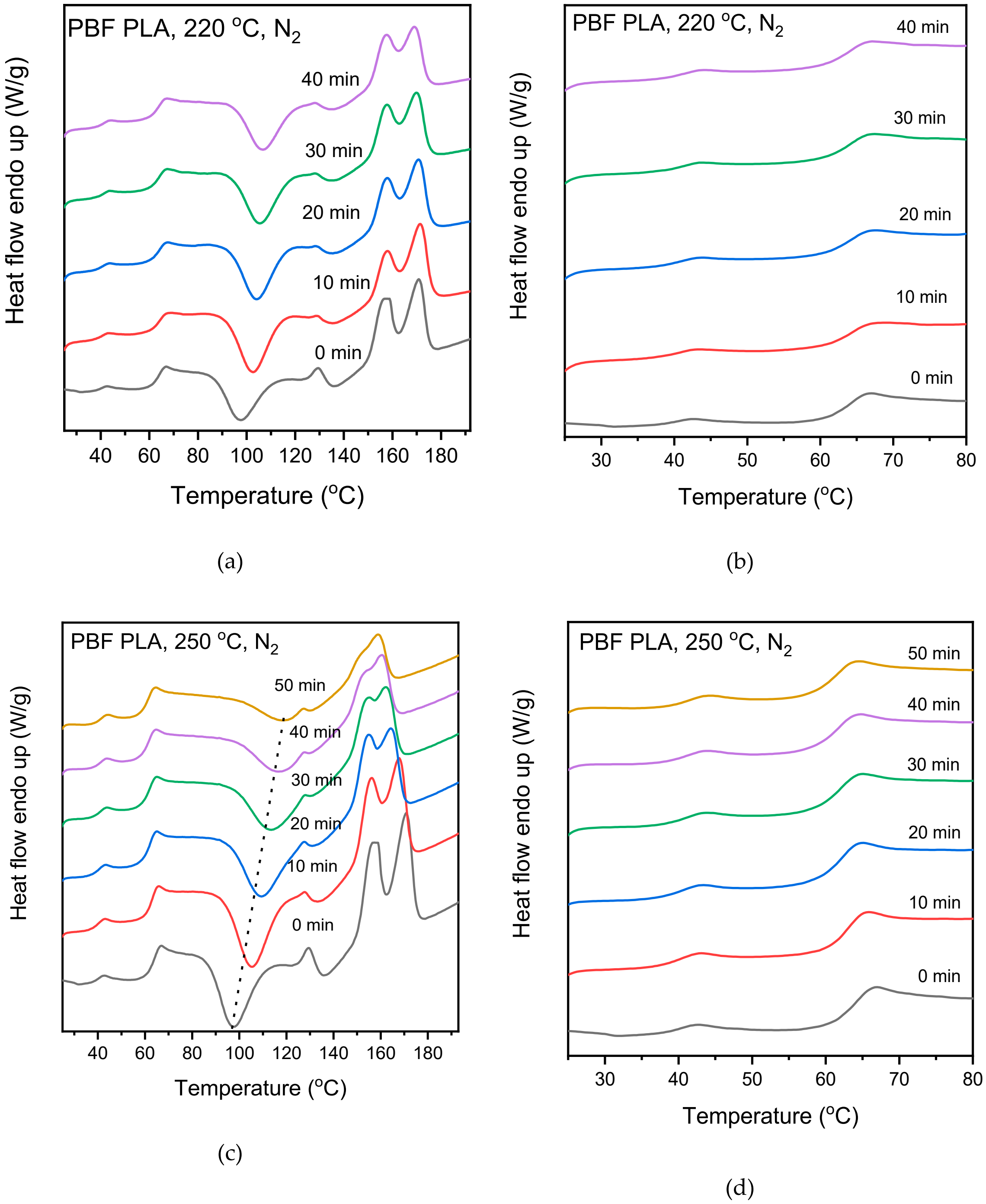

Figure S9. Study of reactive blending with DSC: heating curves after different times, zoom in the T_g region of PBF PLA at 220 °C (a-b) and (c-d) 250 °C.

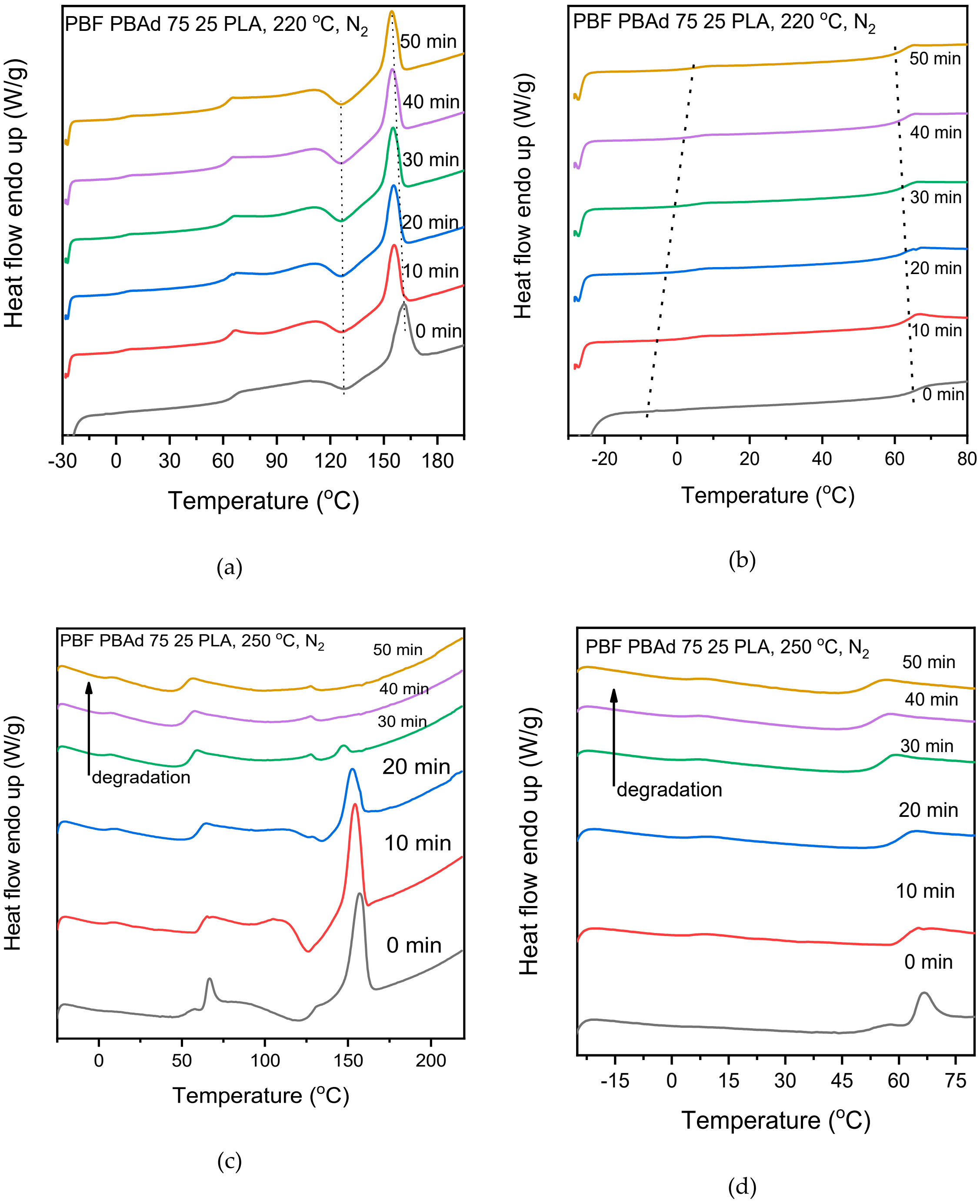

Figure S10. Study of reactive blending with DSC: heating curves after different times, zoom in the T_g region of PBF-PBAd 75 25 PLA at 220 °C (a-b) and (c-d) 250 °C.

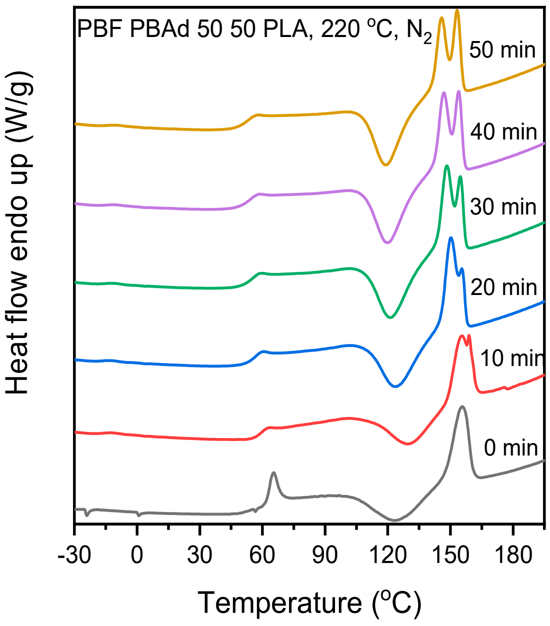

(a)

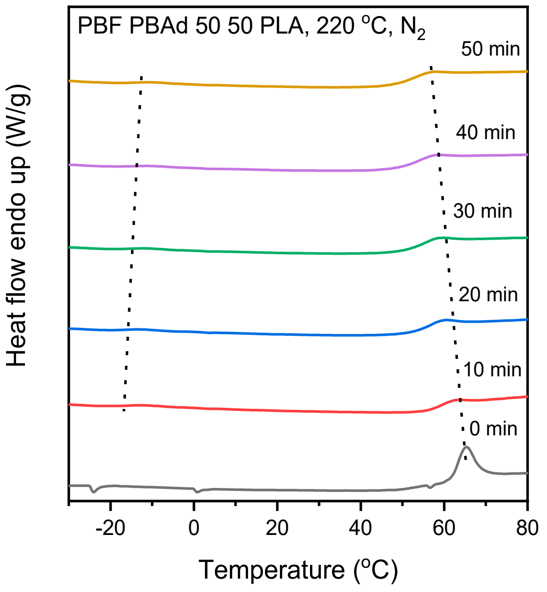

(b)

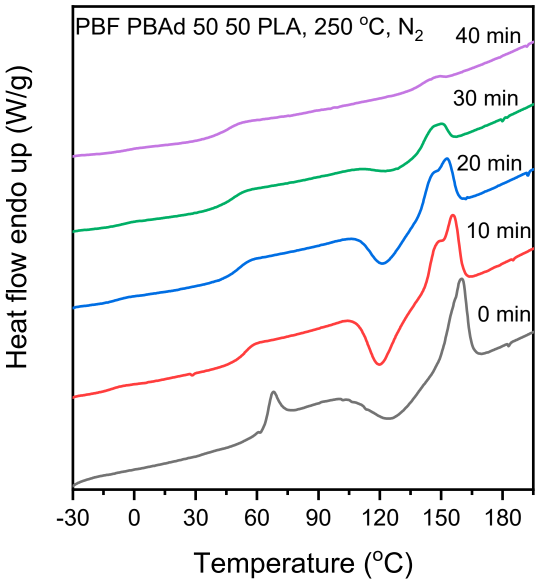

(c)

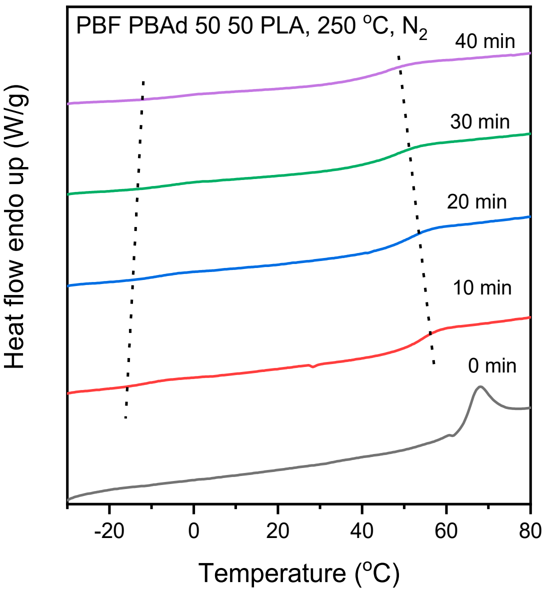

(d)

Figure S11. Study of reactive blending with DSC: heating curves after different times, zoom in the T_g region of PBF-PBAd 50 50 PLA at 220 °C (a-b) and (c-d) 250 °C.

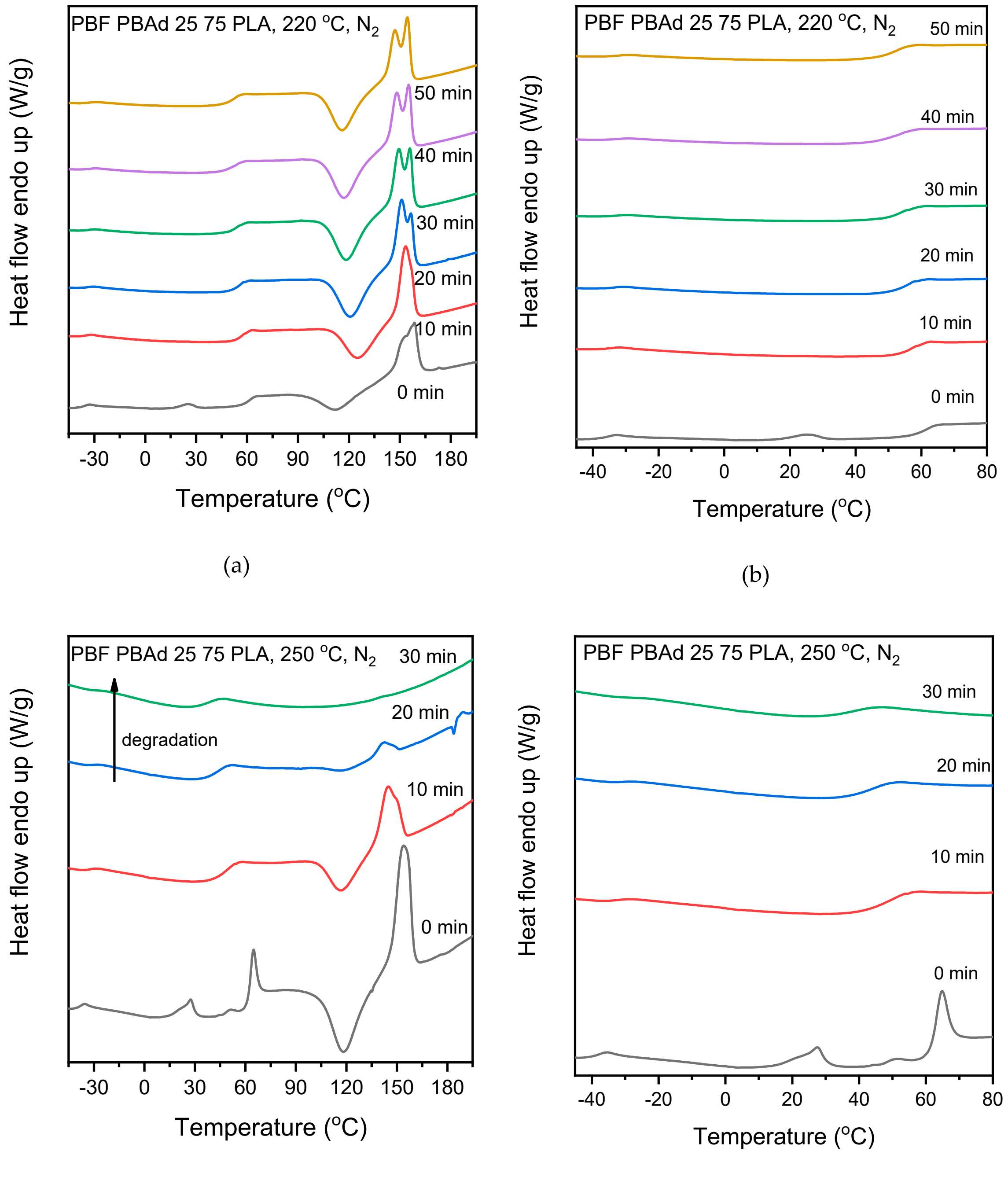

Figure S12. Study of reactive blending with DSC: heating curves after different times, zoom in the T_g region of PBF-PBAd 25 75 PLA at 220 °C (a-b) and (c-d) 250 °C.

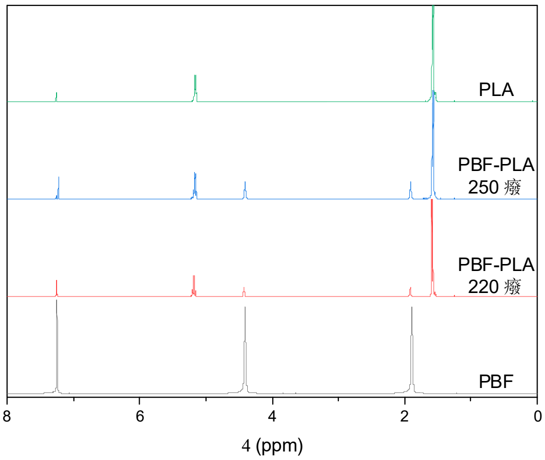

(a)

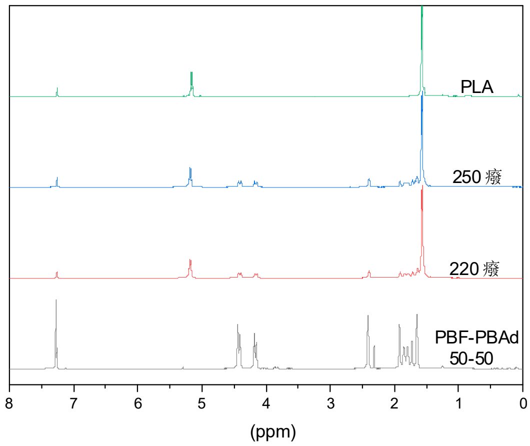

(b)

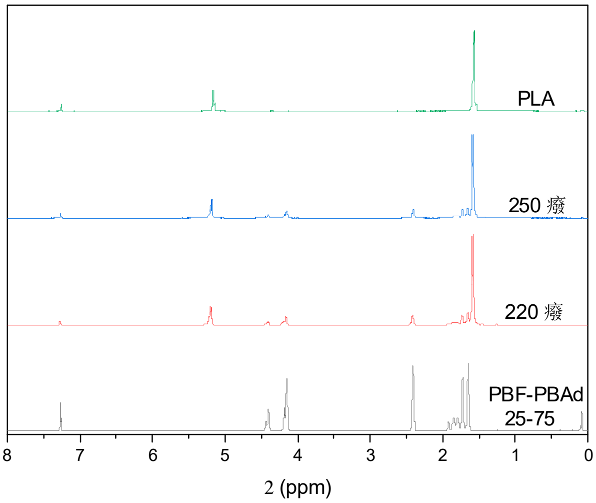

(c)

Figure S13. ^{1}H NMR spectra of (a) PBF PLA, (b) PBF-PBAd 50-50 PLA, (c) PBF-PBAd 25-75 PLA blends, at 220 °C and 250 °C.
